# Meta-analytical transdiagnostic neural correlates in common pediatric psychiatric disorders

**DOI:** 10.1038/s41598-022-08909-3

**Published:** 2022-03-22

**Authors:** Jules R. Dugré, Simon B. Eickhoff, Stéphane Potvin

**Affiliations:** 1 Research Center of the Institut Universitaire en Santé Mentale de Montréal, 7331 Hochelaga, Montreal, QC H1N 3V2 Canada; 2 Department of Psychiatry and Addictology, Faculty of Medicine, University of Montreal, Montreal, Canada; 3 Institute of Neuroscience and Medicine (INM-7), Jülich, Germany; 4 Institute for Systems Neuroscience, Heinrich Heine University, Düsseldorf, Germany

**Keywords:** Social neuroscience, Human behaviour

## Abstract

In the last decades, neuroimaging studies have attempted to unveil the neurobiological markers underlying pediatric psychiatric disorders. Yet, the vast majority of neuroimaging studies still focus on a single nosological category, which limit our understanding of the shared/specific neural correlates between these disorders. Therefore, we aimed to investigate the transdiagnostic neural correlates through a novel and data-driven meta-analytical method. A data-driven meta-analysis was carried out which grouped similar experiments’ topographic map together, irrespectively of nosological categories and task-characteristics. Then, activation likelihood estimation meta-analysis was performed on each group of experiments to extract spatially convergent brain regions. One hundred forty-seven experiments were retrieved (3124 cases compared to 3100 controls): 79 attention-deficit/hyperactivity disorder, 32 conduct/oppositional defiant disorder, 14 anxiety disorders, 22 major depressive disorders. Four significant groups of experiments were observed. Functional characterization suggested that these groups of aberrant brain regions may be implicated internally/externally directed processes, attentional control of affect, somato-motor and visual processes. Furthermore, despite that some differences in rates of studies involving major depressive disorders were noticed, nosological categories were evenly distributed between these four sets of regions. Our results may reflect transdiagnostic neural correlates of pediatric psychiatric disorders, but also underscore the importance of studying pediatric psychiatric disorders simultaneously rather than independently to examine differences between disorders.

## Introduction

Common child psychiatric disorders generally include Attention-deficit/hyperactivity disorder (ADHD), Conduct/Oppositional Defiant Disorder (CD/ODD), anxiety disorders (ANX) and depressive disorders (DEP), which affect approximately 3.4%, 5.7%, 6.5% and 2.6% of children and adolescents in the world, respectively^1^. Indeed, these are the most prevalent disorders in childhood, with age of onset being earlier than other disorders such obsessive compulsive disorder, substance use disorder and schizophrenia^2^. Importantly, evidence suggests that comorbidity between these four pediatric psychiatric disorders is the norm rather than the exception. In fact, about half of children with ADHD, CD/ODD, ANX or DEP will receive an additional psychiatric disorder (comorbid condition) in the following years^3–10^. Although these four diagnostic entities show large comorbidities in children and adolescent, theoretical pathophysiological models taking into account this high level of comorbidity remain largely limited^11^.

Recently, there has been a growing body of literature suggesting that several genetic^12–16^ and environmental risk factors^16–18^ may be non-specific given that they increase the risk for a plurality of psychiatric disorders. Likewise, meta-analyses of structural and functional magnetic resonance imaging studies have shown that adult with psychiatric disorders may share several neurobiological deficits^19–23^. For instance, during cognitive control tasks, transdiagnostic neural signatures in adults with psychiatric disorders (e.g., schizophrenia, bipolar, unipolar depression, anxiety and substance use) may involve the fronto-insular cortex (FIC), the dorsolateral prefrontal cortex and the dorsal anterior cingulate cortex (dACC) to anterior midcingulate/pre-supplementary motor area (aMCC/pre-SMA) and inferior parietal lobule^22^. Similarly, during emotion processing, transdiagnostic features may include deficits in the FIC, amygdala, thalamus and dorso- and ventro-medial PFC^23^. Although some differences have been noticed between patients with and without psychotic disorders^22,23^, the search for shared/specific neurobiological markers is of great interest for our understanding of the psychophysiological mechanisms underlying psychiatric disorders.

In functional neuroimaging literature in childhood/adolescents, studies that aimed to uncover the specific/transdiagnostic neurobiological markers have been scarce. Indeed, a large majority of task-based fMRI studies has focused on a single psychiatric disorder, therefore limiting our ability to identify common/specific neurobiological markers. Additionally, recent transdiagnostic fMRI meta-analyses have excluded disorders which predominantly emerge in childhood/adolescence such as ADHD and CD/ODD^22,23^. Nevertheless, past meta-analyses and reviews on ADHD^24–29^, CD/ODD^30–34^. ANX^35–40^ and DEP^41–48^ seem to indicate qualitatively similar deficits in the anterior insula, medial and lateral prefrontal cortex, the amygdala and anterior to midcingulate cortex. Yet, there is a clear need for meta-analytical evidence of transdiagnostic neural correlates in children and adolescents. Although these results may provide substantial insight for our understanding of transdiagnostic brain alterations, classical meta-analytical approaches are prone to important biases. Indeed, authors’ categorization of groups of interest, categorization of fMRI tasks and the choice of task contrast may significantly alter results. In comparison to the classical meta-analytic approach which seeks to identify dysfunctional brain regions in predefined groups of interest, reverse inference meta-analytical method rather aims to discover main dysfunctional brain regions in which some particular groups may be over/underrepresented. The latter approach may address the limitations of the classic approach by searching for common/specific neural correlates irrespective of the task-characteristics or nosological categories. To our knowledge, only one study has investigated transdiagnostic features across adult samples through a region-of-interest (ROI) reverse-inference meta-analytical method^49^. Given that a single region may be implicated in a wide range of cognitive processes and that co-activation patterns are important in inferring mental processes, the use of a data-driven method (rather than a ROI approach) is crucial to examine transdiagnostic features. Here, we carried out a meta-analysis that primarily aimed to identify groups of aberrant brain regions across pediatric psychiatric disorders using a data-driven meta-analytical method. Results from past meta-analyses on adult samples^22,23^ and disorder-specific meta-analyses and reviews^24–42^ suggest that transdiagnostic features may be expected in FIC (anterior insula/vlPFC), medial and lateral prefrontal and the dorsal anterior and anterior midcingulate cortices. However, considering that deficits in the amygdala is systematically observed in past meta-analyses on adult ANX^36,37^ and DEP^41–48^, but less extensively in CD/ODD^30–34^ and not found in ADHD^24–29^, we hypothesized that the former region would be more closely linked to ANX and DEP than the latter disorders.

## Methods

### Identification of included studies

Our search focused specifically on four diagnostic categories (i.e., ADHD, CD/ODD, ANX, DEP) since they are the most common psychiatric disorders in childhood and they show substantial comorbidity with each other^2–10^. Given that meta-analyses and literature reviews on these disorders have been published recently, we extracted data from their reference lists of ANX^37,38,40^, DEP^41,42,50^, CD/ODD^32^, ADHD^29^. Inclusion criteria were: (1) original manuscript from a peer-reviewed journal, (2) task-based functional MRI studies, (3) use of a whole-brain methodology (i.e., studies using ROIs were excluded) irrespectively of the task constructs, (4) < 18 years old participants meeting criteria for at least one of the following pediatric psychiatric disorder: (a) ADHD; (b) Disruptive disorder (CD/ODD); (c) ANX (i.e., Posttraumatic Stress Disorder, Generalized Anxiety Disorder, Social Anxiety Disorder) and/or (d) Unipolar Major Depressive Disorder. These inclusion criteria were followed to preserve an acceptable level of homogeneity within nosological categories. Effect of the disorder were extracted from fMRI studies, irrespectively of the direction (hypo/hyper activation) of the contrast, to create an aberrant activation map. Two experiments from the same study were considered as distinct if they included two different samples or two different fMRI tasks. Each experiment and sample’s characteristics were manually annoted and categorized. Coordinates of experiments that were reported originally in Talairach stereotaxic space were converted into MNI (Montreal Neurologic Institute) space.

### Neurobiologically-driven meta-analytical procedure

#### Modeled activation & cross-correlation matrix (step 1 & 2)

**Figure 1:**
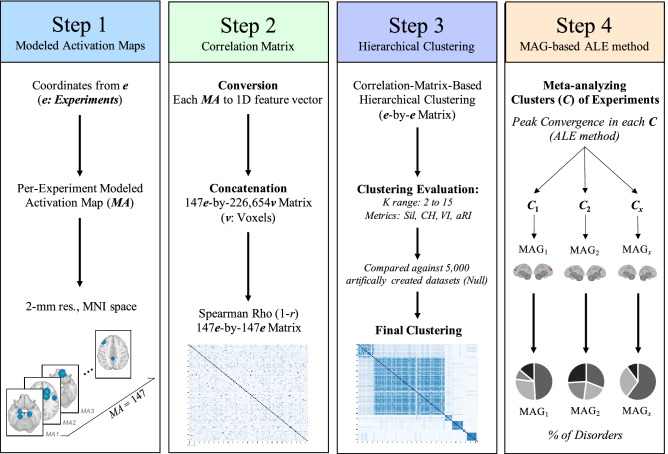
Workflow of the current study. Step 1: Creation of a MA map for each experiment, weighted by sample size. Step 2: Pairwise Spearman Rho correlation was performed between every MA map. Step 3: Clustering analysis was performed on the correlation matrix to extract groups of experiments sharing similar MA map. Step 4: ALE meta-analysis was conducted on experiments within each group. Phenotype assessment was then carried out to investigate under/over-representativeness of disorders, sample and task characteristics across identified groups.

Modeled activation (MA) map was created for each experiment (2 mm^3^ resolution) (Fig. 1, Step 1). Each resulting MA map was converted into a 1D feature vector of voxel values (i.e. 2 mm^3^ grey matter mask in MNI space) and concatenated together to form an experiment (*e*) by voxel matrix (*v*) (147 experiments × 226,654 voxels). Pairwise Spearman’s rank correlation was performed between the 1D feature vector of each experiments to obtain spatial similarity between maps (*e* by *e* symmetric correlation matrix) (Fig. 1, Step 2).

#### Correlation-matrix-based hierarchical clustering (step 3)

In order to extract data-driven groups of experiments that showed similar brain topographic map, we performed a Correlation-Matrix-Based Hierarchical Clustering (CMHC) analysis, as previously used on meta-analytic data^51,52^. The CMHC was carried out using correlation distance (1–*r*) (Fig. 1. Step 2) and average linkage method. We examined the most optimal number of clusters using the silhouette and calinski-harabasz indices, variation of information & adjusted rand index for a range of 2 to 15 clusters^53^ (See Supplementary Material). After having found the final number of meta-analytical grouping (MAGs), solutions with less than 10 experiments were considered as outliers and excluded from further analyses, given that analyses involving < 10 experiments drastically increases the risk that a single experiment drives the results^54^. All these analyses were performed using Scikit-learn (version 0.21.3) in Python (version 3.7.4)^55^.

#### Meta-analytical groupings (Step 4)

Experiments (*e*) within each MAG were then meta-analytically processed (Step 4), using the activation likelihood estimate (ALE) algorithm (GingerALE version 3.0.2)^56,57^. Voxel-wise ALE scores were computed as the union of MA maps, which provide a quantitative assessment of spatial convergence across experiments. These voxel-wise maps were cut off by a cluster-forming threshold. In fact, the size of the supra-threshold clusters was compared against a null distribution of cluster sizes derived from artificially created datasets in which foci were shuffled across experiments, but the other properties of original experiments (e.g., number of foci, uncertainty) were kept^56^. In the current study, we used the following statistical threshold: a voxel-level cluster forming threshold of p < 0.001 and a cluster-level family-wise correction (pFWE < 0.05), with 5000 permutations^54^.

To examine under- and overrepresentations of nosological categories, task and sample characteristics within each MAG, we carried out one-tailed binomial tests comparing their prevalence with their base rate (across all experiments). Main effects of diagnosis, task and sample characteristics between MAGs were investigated through chi-squares (*X*^2^) and Kruskal–Wallis (H) tests. Literature bias was also assessed to compare differences between nosological categories in terms of task and sample characteristics (See Supplementary Material). Finally, for each MAG, we extracted functional characterization using the *Behavioral Analysis plugin* of the Multi-Image Analysis GUI^58^. A z-score higher or equal to 3 is considered significant (i.e., p < 0.05 Bonferroni corrected for multiple comparisons).

## Results

### Identified studies and characteristics

A total of 124 original studies met the inclusion criteria for the meta-analysis, of which 11 involved more than one sample and 8 comprised two or more distinct fMRI task contrasts. This resulted in 147 experiments (1030 foci) involving 3199 cases that were compared to 3024 healthy controls. Mean age of cases was 13.8 years old (SD = 2.25) and the average rate of boys across samples was 71.67%. (see Supplementary Material). Disorder-specific studies showed significant literature bias regarding the choice of neurocognitive task domains, average of sex ratio, and the average of prescribed medication per samples (See Supplementary Table).

### Neurobiologically-driven meta-analysis

#### Clustering solution

Clustering solutions were investigated for a range of *K* = 2–15 MAGs with resampling method (90% subsamples and 5000 iterations). Average of the 5000 iterations metric values for each *K* were plotted. Despite the fact that Calinski-Harabasz exhibited a monotonic behavior (constantly increasing), results from the silhouette index (K = 8), aRI (K = 3 & K = 8) and variation of information (from K = 2–3, from K = 6–7 & K = 7–8) indicated that the solution with 8 MAGs was the most optimal (See Supplementary Fig. 1).

**Figure 2:**
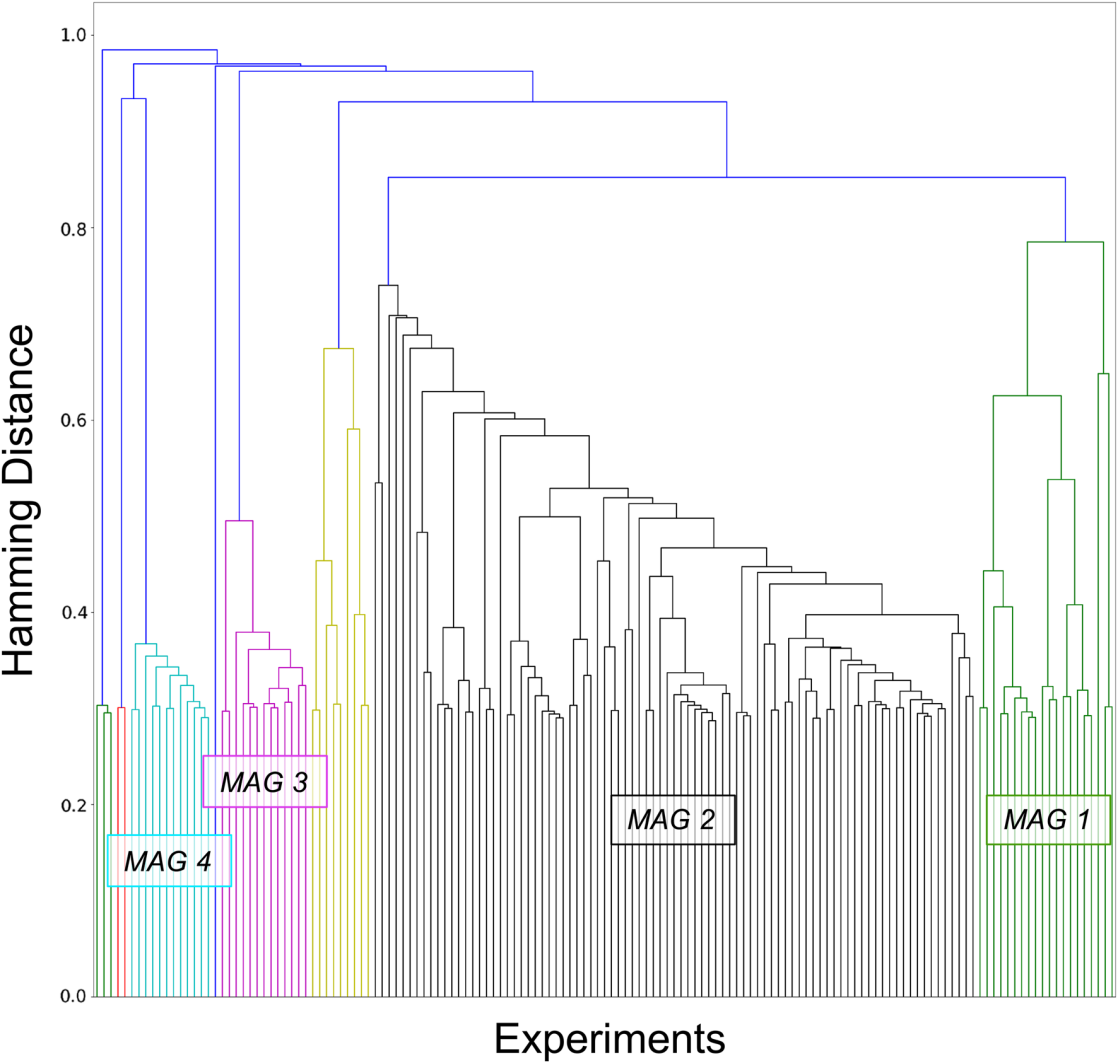
Hierarchical clustering of aberrant activation maps. This dendrogram represents the final hierarchical clustering model which grouped experiment showing similar aberrant activation maps. The 4 significant meta-analytical groupings (MAGs) represented 90.58% of total sample of experiments: MAG1 (green) = 21 experiments and 577 subjects; MAG2 (black) = 87 experiments (1848 subjects); MAG3 = 13 experiments (197 subjects) & MAG4 (cyan) = 12 experiments (278 subjects).

Of the 8 MAGs, 4 comprised less than 10 experiments (n = 8, 3, 2 & 1, respectively). These were excluded from further analyses. The remaining 4 MAGs represented 90.58% of total sample of experiments (133 experiments out of 147): MAG1 (577 subjects, 21 experiments and 120 foci), MAG2 (1848 subjects, 87 experiments, 708 foci), MAG3 (197 subjects, 13 experiments, 52 foci), MAG4 (278 subjects, 12 experiments, 113 foci) (Fig. 2).

#### ALE meta-analysis

**Figure 3:**
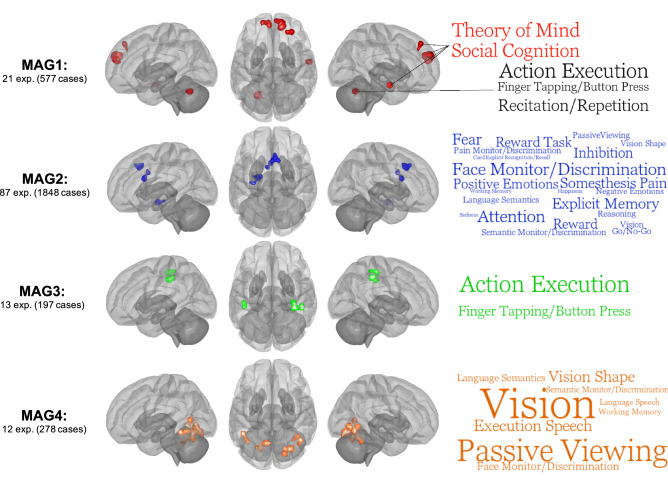
ALE meta-analysis on each significant meta-analytical grouping (MAGs). Images are shown for left hemisphere (lateral), superior view and right hemisphere (lateral) respectively. ALE images were thresholded at p < 0.001 at the voxel-level and pFWE > 0.05. Word clouds were generated using BrainMap database terms (Behavioral Subdomains & Paradigm). Font size represents Z-score associated with the whole MAG (all words are significant p = 0.05 with Bonferroni correction).

As shown in Table 1 and Fig. 3, experiments of the MAG-1 had convergent peaks in the right rostrodorsal dorsomedial PFC (dmPFC) and the left caudal dmPFC (see^59^), the left cerebellum (Lobule VI), the right dorsolateral prefrontal cortex (dlPFC, , Brodmann area (BA) 9/46d^60^) and the middle temporal gyrus (MTG). MAG2 included the right anterior MCC (BA32^61,62^), the left amygdala and the left aMCC (BA24 a’-b’^61,62^). Regarding the MAG3, spatial convergence was found in the right posterior precentral (BA4p) to postcentral gyri (BA2-3), the right supramarginal gyrus and the left postcentral gyrus (BA2) (IntraParietal area2^63^). Finally, spatial map of MAG-4 included occipital/cerebellar regions such as bilateral ventral extrastriate cortex^64^, bilateral fusiform gyrus, bilateral Lobule VI, left calcarine gyrus and right posterior middle/inferior temporal gyrus.

**Table 1:** ALE meta-analysis results of each significant groups of experiments.

MAGs	Clusters	Size (mm^3^)	MNI coordinates			ALE	Cluster breakdown
			X	Y	Z		
MAG1	1	2456	14	46	28	0.0175	R dmPFC (rostrodorsal)
	2	1152	− 16	56	22	0.0154	L dmPFC (caudal)
	3	1096	− 24	− 60	− 28	0.0177	L Cerebellum (Lobule VI)
	4	1048	30	40	46	0.0164	R dlPFC
	5	848	58	− 8	− 18	0.0216	R MTG/STG
MAG2	1	1352	8	18	40	0.0243	R aMCC (Area 32')/pre-SMA
	2	1296	− 20	− 10	− 16	0.0272	L Amygdala
	3	1040	− 2	12	22	0.0321	L dACC (Area 24a'-b')
MAG3	1	976	34	− 26	52	0.0125	R Pre-/Postcentral gyri (Area 2–3 & 4p)
	2	800	46	− 34	44	0.0133	R Supramarginal gyrus (Area 2, PFt)
	3	720	− 42	− 32	42	0.0112	L Postcentral gyrus (Area 2, PFt)
MAG4	1	2336	20	− 78	− 12	0.0172	R Lingual (h0c3v)
	2	1224	− 18	− 66	− 24	0.0159	L Cerebellum (Lobule V1)
	3	968	44	− 58	− 4	0.0168	R pMTG
	4	912	− 44	− 48	− 14	0.0151	L pITG
	5	736	− 8	− 62	6	0.0186	L Calcarine Cortex
	6	728	40	− 52	− 26	0.0155	R Cerebellum (Lobule VI)

*MAG* meta-analytical grouping, *PFC* prefrontal cortex, *dmPFC* dorsomedial PFC, *dlPFC* dorsolateral PFC, *MTG* middle temporal gyrus, *STG* superior temporal gyrus, *aMCC* anterior midcingulate cortex, *pre-SMA* pre-supplementary motor area, *dACC* dorsal anterior cingulate cortex, *IPL* Inferior Parietal Lobule, *SPL* superior parietal lobule, *pMTG* posterior MTG, *pITG* posterior ITG.

#### Functional characterization of MAGs

Functional characterization of MAGs (i.e., MAG-wide & cluster-specific) was performed to examine their relationships with behavioral domains and paradigms of the BrainMap database (see Fig. 3, Supplementary Material):

MAG1: Experiments mainly included response inhibition (7) and reward decision-making tasks (5, e.g., Monetary incentive delay task). Functional characterization using the BrainMap database yielded no significant behavioral/paradigm classes. However, bilateral dmPFC and anterior MTG/STG were positively associated (Z > 3.0) with social cognition/theory of mind, and negatively related (Z < − 3.0) with action execution. Interestingly the left Lobule VI show positive association with action execution and negative relationship with social cognition, whereas dlPFC was related to action inhibition. In sum, this MAG may be characterized by deficits of brain regions subserving social cognition during cognitive & reward decision-making tasks.

MAG2: Experiments within this MAG primarily included task contrasts comprising an emotional component (k = 42) of which 24 used negative emotional stimuli (e.g., facial expression). MAG2 was characterized by a wide range of behavioral subdomains from the BrainMap Database including attention, face monitoring & discrimination and explicit episodic memory. Furthermore, the right aMCC/pre-SMA (Attention) shared similar cognitive domains with left amygdala (Face Monitoring/Discrimination) such as explicit memory, semantic monitoring and positive emotions/reward. Also, the right aMCC/pre-SMA and the left dACC were both associated with the somesthesis pain (monitoring and discrimination) domain. Given these findings, the co-occurrence of the dACC, aMCC/pre-SMA and the amygdala may be involved in stimulus-driven attentional control.

MAG3: Experiments within this MAG included a variety of cognitive and sensorimotor tasks (e.g., finger sequencing, anti-saccade, mental rotation, nback). Using the BrainMap Database, we observed that MAG3 was significantly associated with action execution and finger tapping. Region-specific analyses revealed that the three regions, the right posterior precentral/postcentral, the right SMG and left postcentral, shared action execution, finger tapping and somesthesis behavioral domains. In sum, brain regions of this MAG may encompass sensorimotor/action execution processes.

MAG4: Experiments from the MAG4 mainly included various cognitive tasks (10). Functional characterization using the BrainMap database revealed significant associations with vision, passive viewing and speech execution. Region-specific analyses revealed that all but the calcarine were significantly related to vision. Furthermore, the right pMTG/ITG, the left pITG/FF and the right lobule VI shared face monitoring/discrimination, passive viewing, vision shape and covert naming domains. In short, MAG4 may reflect co-occurrent deficits in brain regions involved in visual processing during cognitive tasks.

#### Phenotype assessment 1: nosological categories

MAG1 was less likely to include DEP samples (X_2_ = 4.16, p = 0.041), compared to all the other MAGs (Table 2). Indeed, proportions of DEP samples in MAG1 was significantly lower than its base rate (0% versus 15.00%, one-tailed p = 0.028). Taking into account the between-disorder literature bias revealed that the lower rates of DEP samples in MAG1 were replicated when restricting experiments to those using an emotional task contrast and mixed sex samples (Supplementary Material).

**Table 2:** Characteristics of Experiments across meta-analytical groupings.

Characteristics	Total (n = 147)		MAG1 (k = 21)		MAG2 (k = 87)		MAG3 (k = 13)		MAG4 (k = 12)	
	n	%	n	%	n	%	n	%	n	%
**Nosological categories**										
ADHD	79	53.7%	14	66.7%	43	49.4%	8	61.5%	8	66.7%
CD	32	21.8%	4	19.0%	17	19.5%	4	30.8%	3	25.0%
ANX	14	9.5%	3	14.3%	9	10.3%	0	0.0%	1	8.3%
DEP	22	15.0%	0*†	0.0%	18†	20.7%	1	7.7%	0	0.0%
**Task-contrast domain**										
Cognitive	88	59.9%	10	47.6%	53	60.9%	9	69.2%	10	83.3%
Response Inhibition	44	29.9%	7	33.3%	29	33.3%	3	23.1%	4	33.3%
Attention	23	15.6%	1	4.8%	13	14.9%	3	23.1%	3	25.0%
Emotion	71	48.3%	12	57.1%	42	48.3%	3*	23.1%	5	41.7%
Positive	17	11.6%	6*†	28.6%	7*	8.0%	0	0.0%	1	8.3%
Negative	37	25.2%	4	19.0%	24	27.6%	1	7.7%	2	16.7%
Both	16	10.9%	2	9.5%	10	11.5%	2	15.4%	2	16.7%
**Sample characteristics**										
Medication-Naïve	61	41.5%	14†	66.7%	40	46.0%	6	46.2%	5	41.7%
Average Med per sample	–	26.7%	–	35.2%	–	26.3%	–	19.4%	–	20.9%
Mixed Sex Sample	95	64.6%	12	57.1%	60	69.0%	7	53.8%	8	66.7%
Average Boys per Sample	–	71.7%	–	77.6%	–	71.4%	–	76.1%	–	61.1%

*Represents significant difference compared to its base rate (one-tailed p < 0.05). † Represents significant differences between MAGs (p < 0.05).

Additionally, MAG2 had more DEP samples than other MAGs (X_2_ = 8.43, p = 0.004). However, compared to its base rate, proportion of DEP samples was not significantly overrepresented in MAG2 (20.7% versus 15.0%, one-tailed p = 0.123). After taking into account the between-disorder literature bias, we observed that the higher rates of DEP samples in MAG2 was replicated when restricting experiments to those using a cognitive task contrast or an emotional task contrast but also in experiments with only medication naïve sample and mixed sex samples (Supplementary Material). Graphical representation of probabilities of aberrant MAG per disorder class can be found in Supplementary Fig. 2.

#### Phenotype assessment 2: task & sample characteristics

We observed that the rate of experiments within the MAG1 that included a positive emotional stimulus was higher than other MAGs (X_2_ = 8.62, p = 0.003) (Table 2), and significantly overrepresented compared to its base rate (28.6% versus 11.6%, one-tailed p = 0.028).

Additionally, experiments in MAG2 were less likely to include positive emotional task contrast (X_2_ = 3.97, p = 0.046) and marginally associated with greater experiments with negative emotion task contrast (X_2_ = 3.31, p = 0.069), compared to other MAGs (Table 2). However, proportions of these task domains were not statistically different than their base rates.

MAG3 had significantly lower rate of general emotional stimuli compared to other MAGs (X_2_ = 4.20, p = 0.040), which was marginally lower compared to its base rate (37.5% versus 48.3%, one-tailed p = 0.059). Other characteristics did not reach statistical significance, compared to their base rates.

Although MAG1 had a significantly higher rate of medication-naïve subjects, compared to its base rate (one-tailed p = 0.018), MAGs did not differ in rates of experiments with medication-naïve samples (X^2^ = 2.25, p = 0.522) and average rate of prescribed medication (Kruskal-Wall H = 2.74, p = 0.433). No differences were observed concerning the rate of mixed sex samples (X^2^ = 1.90, p = 0.594) and the average rate of boys in samples (Kruskal-Wall H = 2.40, p = 0.493).

## Discussion

The current meta-analysis was carried out to examine the shared and/or specific neural correlates of pediatric psychiatric disorders (ADHD, CD/ODD, ANX & DEP). To do so, we used a novel data-driven meta-analytical method that aimed to extract groups of experiments which show similar brain topographic maps. We identified 4 significant MAGs, which comprised co-occurrent deficits in brain regions that may share features with (1) internally/externally directed processes; (2) attentional control of emotions, (3) action execution and (4) visual processes. More importantly, compared to their base rate, we found underrepresentation of DEP samples in MAG1 and overrepresentation in MAG2. However, no other significant differences were found in nosological categories between MAGs nor by considering their base rates, suggesting potential transdiagnostic correlates.

MAG1 included bilateral dmPFC, dlPFC, MTG/STG and Lobule VI. More precisely, we observed that dmPFC-MTG/STG were involved in social cognitions, whereas dlPFC and Lobule VI were characterized as action inhibition and execution, respectively. We also found main task-effect of the utilization of a positive emotional stimulus. Interestingly, findings suggest that during cognitively demanding tasks, brain regions involved in internally directed processes (e.g., dmPFC & anterior MTG/STG) flexibly shifts their activity to enable goal-directed processes (e.g., dlPFC & Lobule VI)^65–67^. Given these data, deficits in brain regions involved in MAG1 may reflect a failure to disengage internal processes at the cost of goal-directed processes^68^. Interestingly, our results suggest that these co-occurrent deficits (i.e., dmPFC, dlPFC, Lobule VI and MTG/STG) are less likely to be reported in that DEP samples. However, some studies have shown deficits in fronto-parietal and DMN regions in internalizing disorders^69,70^. Given that these findings were observed in adult samples and that anticorrelation between these processes varies from childhood from adulthood^71^, it is possible that these deficits may be observed in adulthood but not childhood DEP. Also, the DEP samples did not frequently report using positive emotional stimuli (k = 3 out of 22), which was found to be the main task-characteristic of MAG1. This may suggest that the lack of relationship between MAG1 and DEP may be explained by task differences. Considering the small sample size involved, we cannot completely rule out the possibility of MAG1 deficits in children with DEP.

The largest MAG (MAG2, k = 87) was constituted of the aMCC/pre-SMA, amygdala and dACC. This MAG was mainly characterized by attention, face monitoring and explicit episodic memory, using the BrainMap database. We found higher rates of DEP samples, compared to other MAGs, which concur with past meta-analytical evidence consistently showing aberrant activation in these particular regions during negative emotional tasks in adults with major depression^43–48^. However, no effect was observed in ANX samples, potentially due to the limited sample size. Nonetheless, deficits in these regions were also observed across adult ANX & DEP samples, during negative emotion processing^21,23,72^. Additionally, we observed a marginally significant association between this MAG and negative emotional stimuli, indicating a possible task-effect. Although rates of ADHD (≈50%), CD/ODD (≈20%) did not differ between other MAGs, evidence suggests that these disorders may also show deficits in MAG2 regions, particularly during emotion processing tasks^32,73^ which correlates with general psychopathology score^69,74,75^. In sum, this MAG may reflect general deficits in emotional lability, inherent to DEP, yet frequently observed in children/adolescent with ADHD^76^ and/or CD^77^.

We also found deficits in brain regions (e.g., pre- and postcentral gyrus) subserving action execution/finger tapping tasks (MAG3). This MAG was less likely to comprise emotional tasks, which is consistent with the fact that emotional tasks usually require less motor execution. Interestingly, deficits in similar regions (i.e., somato-motor network) were also observed in a recent study showing significant transdiagnostic association with general maladaptive functionality^78^. Although deficits in these regions are currently not well understood, sensory deficits such as tactile perception and body awareness are often reported in children with pediatric psychiatric disorders^79–83^. It is thus possible that abnormalities in MAG3 may reflect deficits in tactile perception, crucial for accurate performance of purposeful movements^84^ such as in cognitive tasks.

Finally, we found evidence of early processing deficits across disorders (MAG4). Recent studies have shown replicable structural alterations in brain regions spanning this MAG. In fact, the authors demonstrated, through two different samples comprising 1246^85^ and 875^86^ subjects, that the general psychopathology factor score was associated with deficits in occipital and cerebellum regions. These regions are implicated in variety of visual functions such as detecting relevant changes in the environment (e.g., visual oddball)^87,88^. Thus, MAG4 may mirror several dysfunctional processes in early visual processing, including gazing at task-irrelevant stimuli. For example, during face-emotion tasks, the number and duration of fixation to the eye regions have been reported to be significantly lower in ADHD with and without CD^89^, in childhood psychopathic traits^90^, in ODD/CD^91–93^, anxiety disorders^94–97^) and depression^98,99^. Likewise, deficits in the ability to filter out irrelevant stimuli are also observed in continuous performance test^100^ and visual search tasks^99^ in these populations.

Examining transdiagnostic features using the classical meta-analytic approach yielded aberrant activation in the aMCC/pre-SMA (see Supplementary Material). Furthermore, we found that externalizing disorders (i.e., ADHD, CD/ODD) were associated with deficits in the pre-SMA, whereas internalizing disorders (i.e., ANX, DEP) yielded aberrant activity in the dorsal/perigenual ACC. Interestingly, deficits in these regions were also found to be transdiagnostic neurobiological features in adult samples (dACC & aMCC^22^). It nonetheless remains unknown whether these transdiagnostic features may be due to a common vulnerability (e.g., shared risk factors) or the presence of cross-cutting criteria (e.g., impulsivity, neuroticism), which should be tackled in the future. Also, we observed no significant peak convergence across each of the disorder-specific meta-analysis. This lack of convergence concurs with results from recent meta-analyses which revealed similar results in CD/ODD, ADHD and DEP, using a somewhat conservative threshold (p < 0.001, cFWE < 0.05)^29,32,101^. Despite that this lack of convergence might have been attributable to between-study differences (e.g., stimulus, sex effect, statistical threshold, sample size), one possibility that deserves careful attention is the within-disorder heterogeneity. Indeed, it is generally well accepted that DSM-derived categories comprise subfactors that are characterized by different psychological processes^102–107^. Thus, this heterogeneity in criteria substantially increases the risk of finding distinct set of symptoms while still meeting the diagnostic threshold (from 42 [GAD] to 116,200 [ADHD] theoretical set of criteria^9,108^. Therefore, we could not rule out the possibility that increasing the sample size in meta-analyses, which also increase the between-sample heterogeneity, may reduce the ability to detect robust findings.

## Limitations

First, included studies were extracted from previous and recent published meta-analyses and literature reviews. Despite that several references were used for each disorder, a systematic search following the PRISMA protocol may have allowed us to identify other studies. Also, we performed cluster analysis across pediatric psychiatric disorders and fMRI paradigms. Since there were limited data available to perform domain-specific analyses, it is possible that our results may have been altered by literature bias (see Supplementary Material) concerning the use of particular neurocognitive task domains per diagnosis category. However, subanalyses were carried out to examine these confounding effects. Second, the limited sample size in the meta-analysis, such as in the ANX sample (k = 14) may have explained the null findings in *classical* disorder-specific meta-analysis and the lack of over/underrepresentation across MAGs and neurocognitive domains. Hence, increasing the number of studies may permit us to detect such differences and unveil more precise aberrant co-activation maps, crucial for understanding transdiagnostic correlates across pediatric psychiatric disorders. Furthermore, we did not provide additional subanalyses on hypo- versushyper-activations across disorders, as the goal of this study was to identify aberrant co-activation maps across disorders and due to the limited number of studies in the case of anxiety disorders. As doing so would have been more optimal, future studies are encouraged to use these maps to examine whether disorders may differ in terms of hypo/hyper-activations. In this meta-analysis, we focused on four main nosological categories to identify shared neural correlates that may reflect their high comorbidities in childhood/adolescence. Future meta-analysis may consider including other disorders such as autism, bipolar depression and phobias to examine differences in neural markers. Finally, we used hierarchical clustering with spearman correlation as distance measure and average linkage algorithm. Although these parameters are frequently utilized in studies using similar meta-analytical approaches, it is possible that the most optimal set of parameters would have been specific to our study.

## Conclusions

We observed transdiagnostic neural correlates across common pediatric psychiatric disorders. The identified groups of co-occurrent deficits shared features with internally/externally directed processes, emotional lability, somato-motor & visual processes. We found that DEP samples were less likely to display aberrant co-activation map involving internally/externally directed processes, but more likely to exhibit deficits in brain regions implicated in attentional control of emotions. Also, these MAGs did not specifically fit particular neurocognitive domains, but rather involved multiple subprocesses (e.g., Self-reflective & Execution/Inhibition; Threat system & Attentional Control). Our results underscore the need for including several psychiatric samples in fMRI studies rather than a single nosological category. As our results indicate shared deficits that could underlie the high rates of comorbidity among children with psychiatric disorders, meta-analyzing between-disorder contrasts, at a study-level, is of great importance to unveil disorder-specific neurobiological markers. Future studies are encouraged to examine how dysfunctions in MAGs may predict worsened outcomes in adulthood, as well as tackling the heterogeneity within psychiatric disorders.

## Supplementary Information

Supplementary Information.

## Data Availability

The dataset analyzed in the current study is available from the corresponding author upon reasonable request.
