# Supplementary material for: Meta-analytical transdiagnostic neural correlates in common pediatric psychiatric disorders
Source: Sci Rep. 2022 Mar 22;12:4909. doi: 10.1038/s41598-022-08909-3 (PMC8941086; doi:10.1038/s41598-022-08909-3)
Supplement: Supplementary file 1 — Supplementary Information. [file 41598_2022_8909_MOESM1_ESM.pdf]

– SUPPLEMENTARY MATERIAL –

Meta-analytical Transdiagnostic Neural Correlates in common Pediatric Psychiatric Disorders

Jules R. Dugré, MSc [1,2], Simon B. Eickhoff [3,4] & Stéphane Potvin, PhD [1,2]

[1] Research Center of the Institut Universitaire en Santé Mentale de Montréal, Montreal, Canada
[2] Department of Psychiatry and Addictology, Faculty of medicine, University of Montreal, Montreal, Canada
[3] Research Centre Jülich, Institute of Neuroscience and Medicine (INM-7), Jülich, Germany.
[4] Institute for Systems Neuroscience, Heinrich Heine University, Düsseldorf, Germany.

Corresponding authors

Jules Roger Dugré, PhD Candidate & Stéphane Potvin, PhD;
Research Center of the Institut Universitaire en Santé Mentale de Montréal; 7331 Hochelaga, Montreal, Quebec, Canada; H1N 3V2; Phone: 514-251-4015;
Email: jules.dugre@umontreal.ca / stephane.potvin@umontreal.ca

Table of Contents

Supplementary Table 1. Included studies: Sample characteristics and fMRI tasks 3

Supplementary Table 2. Included studies: Medication and task-characteristics 6

Additional Information about the Correlation-Matrix Hierarchical Clustering 9

Supplementary Figure 1. Metrics computed for K=2 to 15 clustering solutions 11

Supplementary Figure 2. Probability of Aberrant MAG by disorder class 12

Supplementary Figure 3. Results from the forward (classical) ALE meta-analyses 13

Supplementary Table 3. Results from disorder-specific meta-analyses (p<0.001 unc.) 14

Supplementary Table 4. List of fMRI tasks per MAGs 15

Supplementary Phenotype Assessment: Literature Bias & Subanalyses 18

Supplementary Table 5. Disorder-specific sample and task characteristics 19

Supplementary Table 6. Disorder-specific comorbidities 19

Supplementary Table 7. Disorder-specific comorbidities per MAGs 20

Disorder-Specific ALE Meta-analyses 21

REFERENCES 22

Supplementary Table 1. Included studies: Sample characteristics and fMRI task.

Primary Diagnosis	First Author, Year	Experimental Task	TD (N=)	CASES (N=)	Male (%)	Mean Age
ADHD	Braet et al. 2011 (1)	Go-No/Go	38	20	0.85	14.1
ADHD	Bédard et al. 2014 (2)	N-Back	21	24	0.88	12.4
ADHD	Bollmann et al. 2017 (3)	Spatial Working Memory Task	14	12	0.47	10.9
ADHD	Booth et al. 2005 (4)	Go-No/Go	12	12	0.67	11.0
ADHD	Cao et al. 2008 (5)	Cued Target Detection	13	12	1.00	13.4
ADHD	Cerullo et al. 2009 (6)	Continuous Performance Task	13	10	0.70	14.0
ADHD	Chou et al. 2015$_1$ (7)	Stroop Task	20	25	0.85	10.5
ADHD	Chou et al. 2015$_2$ (7)	Stroop Task	20	25	0.85	10.5
ADHD	Christakou et al. 2013 (8)	Sustained Attention Task	20	20	1.00	14.0
ADHD	Durston et al. 2006 (9)	Go-No/Go	11	11	1.00	13.9
ADHD	Durston et al. 2007$_1$ (10)	Go-No/Go	12	12	1.00	14.9
ADHD	Durston et al. 2007$_2$ (10)	Go-No/Go	12	12	1.00	14.9
ADHD	Ercan et al. 2016 (11)	Go-No/Go	100	50	0.58	11.0
ADHD	Fan et al. 2014 (12)	Stroop Task	23	25	0.92	10.9
ADHD	Hart et al. 2014 (13)	Stop Task	20	20	1.00	12.1
ADHD	Hart et al. 2014 (14)	Stop Task	30	30	1.00	13.9
ADHD	Hauser et al. 2014 (15)	Reversal Learning Task	20	20	0.65	14.6
ADHD	Hwang et al. 2015 (16)	Stroop Paradigm	35	26	0.65	14.5
ADHD	Iannaccone et al. 2015$_1$ (17)	Speeded Flanker Task	18	18	0.61	14.5
ADHD	Iannaccone et al. 2015$_2$ (17)	Speeded Flanker Task	18	18	0.61	14.5
ADHD	Janssen et al. 2015 (18)	Stop-Signal Task	17	21	0.91	10.6
ADHD	Kohls et al. 2014 (19)	Rewarded Go-No/Go	17	16	1.00	14.5
ADHD	Konrad et al. 2006 (20)	Attention Network Test	16	16	1.00	10.2
ADHD	Krauel et al. 2007 (21)	Recognition Test	12	12	1.00	14.5
ADHD	Li et al. 2014 (22)	N-Back	27	33	1.00	9.9
ADHD	Ma et al. 2012 (23)	Go-No/Go	15	15	0.53	9.9
ADHD	Ma et al. 2016 (24)	Rewarded Stroop Task	33	25	0.76	15.4
ADHD	Massat et al. 2012 (25)	N-Back	14	19	0.47	10.8
ADHD	Massat et al. 2018 (26)	Stop Signal Task	19	18	0.44	10.6
ADHD	Metin et al. 2017 (27)	Spatial Attention Paradigm	16	19	0.74	10.32
ADHD	Mostofsky et al. 2006 (28)	Finger Sequencing Paradigm	11	11	0.73	10.4
ADHD	Norman et al. 2017 (29)	Sustained Attention Task	20	20	1.00	15.0
ADHD	Norman et al. 2018 (30)	Iowa Gambling Task	20	16	1.00	14.6
ADHD	O'Conaill et al. 2015 (31)	Visual Search Task	21	19	0.90	11.9
ADHD	Orinstein et al. 2014 (32)	Auditory Oddball Attention Task	20	18	0.78	15.2
ADHD	Passarotti et al. 2010 (33)	N-Back	19	14	0.64	13.0
ADHD	Passarotti et al. 2010 (34)	Emotional Valence Stroop Task	14	15	0.80	12.9
ADHD	Passarotti et al. 2010 (35)	Response Inhibition Task	15	11	0.55	13.1
ADHD	Poissant et al. 2016 (A) (36)	Pictures Story	12	7	0.00	11.0
ADHD	Poissant et al. 2016 (B) (36)	Pictures Story	9	16	1.00	11.0
ADHD	Posner et al. 2011 (37)	Subliminal Fearful Faces	15	15	0.87	13.5
ADHD	Posner et al. 2011 (38)	Emotional Stroop Task	15	15	0.87	13.5
ADHD	Rubia et al. 2005 (39)	Stop Task	22	16	1.00	13.0
ADHD	Rubia et al. 2008 (40)	Stop Task	20	20	1.00	13.2
ADHD	Rubia et al. 2009 (41)	Reward Continuous Performance	16	18	1.00	13.3
ADHD	Rubia et al. 2009 (42)	Simon Task	20	20	1.00	13.2
ADHD	Rubia et al. 2009 (43)	Delay Discounting Task	10	10	1.00	14.0
ADHD	Rubia et al. 2010 (44)	Visual–Spatial Switch Task	20	14	1.00	13.3
ADHD	Rubia et al. 2010$_1$ (45)	Stop Task	20	18	1.00	14.5

ADHD	Rubia et al. 2010$_2$ (45)	Switch Task	-	12	1.00	13.7
ADHD	Rubia et al. 2011 (46)	Simon Task	20	18	1.00	14.3
ADHD	Schwarz et al. 2015 (47)	Anti-Saccades	11	11	0.64	10.4
ADHD	Sheridan et al. 2007 (48)	Match-to-Sample Task	10	10	0.00	15.2
ADHD	Silk et al. 2008 (49)	Raven's Progressive Matrices	12	12	1.00	11.2
ADHD	Siniatchkin et al. 2012 (50)	Go/NoGo	27	17	0.83	9.3
ADHD	Smith et al. 2008 (51)	Time Discrimination	17	21	1.00	12.8
ADHD	Spinelli et al. 2011 (52)	Go-No/Go	17	13	0.69	10.6
ADHD	Spinelli et al. 2011 (53)	Go-No/Go	17	13	0.69	10.6
ADHD	Stevens et al. 2017$_1$ (A) (54)	Monetary Incentive Delay	134	40	0.78	14.8
ADHD	Stevens et al. 2017$_2$ (A) (54)	Monetary Incentive Delay	-	-	0.78	14.8
ADHD	Stevens et al. 2017$_1$ (B) (54)	Monetary Incentive Delay	-	31	0.84	15.0
ADHD	Stevens et al. 2017$_2$ (B) (54)	Monetary Incentive Delay	-	-	0.84	15.0
ADHD	Stevens et al. 2017 (C) (54)	Monetary Incentive Delay	-	46	0.80	15.1
ADHD	Tamm et al. 2004 (55)	Go-No/Go	12	10	1.00	16.0
ADHD	Tamm et al. 2006 (56)	Oddball Task	12	14	1.00	15.6
ADHD	Tamm et al. 2012 (57)	Fluid Reasoning Task	10	12	0.54	9.0
ADHD	Tegelbeckers et al. 2015 (58)	Visual Oddball Task	19	19	1.00	13.3
ADHD	Vance et al. 2007 (59)	Mental Rotation Task	12	12	1.00	11.1
ADHD	van Ewijk et al. 2015 (60)	Spatial span task	103	109	0.60	17.1
ADHD	van Rooij et al. 2015 (61)	Stop-Signal Task	111	185	0.70	17.3
ADHD	Vetter et al. 2018 (62)	perceptual discrimination task	25	25	1.00	14.3
ADHD	Vloet et al. 2010 (63)	Spatial Stimulus–Response	14	14	1.00	11.3
ADHD	von Rhein et al. 2015 (64)	monetary incentive delay task	108	150	0.70	17.7
ADHD	Wang et al. 2013 (65)	Continuous Performance Task	31	28	0.89	9.6
ADHD	Hegarty et al. 2021 (66)	Stop-Signal Task	30	30	0.67	10.1
ADHD	Finger et al., 2008 (67)	Probabilistic Reversal Task	14	14	0.71	13.4
ADHD	Gatzke-Kopp et al. 2009 (68)	Monetary Incentive task	11	19	1.00	13.6
ADHD	Bubenzer-Busch et al. 2016$_1$ (69)	Point Subtraction Aggression	27	27	1.00	10.9
ADHD	Bubenzer-Busch et al. 2016$_2$ (69)	Point Subtraction Aggression	27	27	1.00	10.9
CD/ODD	Banich et al. 2007 (70)	Stroop Task	12	12	1.00	16.7
CD/ODD	Bjork et al. 2010 (71)	Monetary Incentive Delay Task	12	12	1.00	15.4
CD/ODD	Crowley et al. 2010$_1$ (72)	Colorado Balloon Game	20	20	1.00	16.5
CD/ODD	Crowley et al. 2010$_2$ (72)	Colorado Balloon Game	20	20	1.00	16.5
CD/ODD	Dong et al. 2017 (73)	Facial Expression (Pain)	36	30	1.00	15.1
CD/ODD	Fairchild et al. 2014 (74)	Emotional Face Task	20	20	0.00	16.9
CD/ODD	Fehlbaum et al. 2018 (75)	Affective Stroop Task	39	39	0.74	15.9
CD/ODD	Finger et al. 2011$_1$ (76)	Probabilistic Reversal Task	15	15	0.60	14.1
CD/ODD	Finger et al. 2011$_2$ (76)	Probabilistic Reversal Task	15	15	0.60	14.1
CD/ODD	Herpertz et al. 2008 (77)	Passive-Viewing (Emotional)	22	22	1.00	14.7
CD/ODD	Hwang et al. 2016 (A) (78)	Affective Stroop Task	28	17	0.71	14.8
CD/ODD	Hwang et al. 2016 (B) (78)	Affective Stroop Task	-	18	0.56	14.6
CD/ODD	Klapwijk et al., 2016a (79)	Empathic Emotional Face Task	33	23	1.00	16.6
CD/ODD	Klapwijk et al., 2016b (80)	Dictator Game	33	32	1.00	16.8
CD/ODD	Marsh et al., 2011 (81)	Implicit Association Test	14	14	0.57	14.4
CD/ODD	Marsh et al., 2013 (82)	Empathic Situation of Pain Task	21	14	0.54	15.4
CD/ODD	Passamonti et al., 2010 (A) (83)	Emotional Face Task	40	27	1.00	17.7
CD/ODD	Passamonti et al., 2010 (B) (83)	Emotional Face Task	-	25	1.00	17.1
CD/ODD	Rubia et al. 2008 (40)	Stop Task	20	13	1.00	13.0
CD/ODD	Rubia et al. 2009 (41)	Reward Continuous Performance	16	14	1.00	12.8
CD/ODD	Rubia et al. 2009 (42)	Simon Task	20	13	1.00	12.9
CD/ODD	Rubia et al. 2010 (44)	Visual–Spatial Switch Task	20	14	1.00	12.6

CD/ODD	Schwenck et al., 2017 (84)	monetary gambling task	24	19	1.00	13.8
CD/ODD	Thornton et al., 2017 (85)	Animacy Attention Task	20	29	0.69	14.7
CD/ODD	White et al., 2012a (86)	Eye Gaze Task	19	17	0.76	15.5
CD/ODD	White et al., 2012b (87)	Emotion-Attention Bars task	17	15	0.80	15.7
CD/ODD	White et al., 2013_1 (88)	Passive Avoidance Task	18	20	0.82	15.2
CD/ODD	White et al., 2013_2 (88)	Passive Avoidance Task	18	20	0.82	15.2
CD/ODD	White et al., 2014 (89)	Doors Task	15	15	0.73	14.4
CD/ODD	Zhang et al., 2015 (90)	Go/Stop Task	40	29	1.00	15.1
CD/ODD	Zhu et al. 2014 (91)	Go/Stop Task	10	11	1.00	11.5
CD/ODD	Raschle et al. 2019 (92)	emotion-regulation task	29	30	0.00	16.3
ANX	Yang et al. 2004 (93)	Emotional Perception	6	5	0.20	13.0
ANX	Carrion et al. 2008 (94)	Go-No/Go	14	16	0.56	13.7
ANX	Keding-Herringa et al. 2016 (95)	Dynamic Face Task	25	28	0.35	14.3
ANX	Hart et al. 2018 (96)	emotion discrimination task	27	20	0.70	17.5
ANX	Carlisi et al. 2017 (97)	face-attention paradigm	19	14	0.29	14.1
ANX	Gold et al. 2020 (98)	threat conditioning	47	53	0.45	12.8
ANX	Thomas et al. 2001 (99)	Passive-Viewing Emotional Face	12	12	0.58	12.8
ANX	Monk et al. 2006 (100)	Emotional Face probe detection	15	18	0.47	12.3
ANX	Monk et al. 2008 (101)	Emotional Face visual probe task	12	17	0.65	13.1
ANX	Strawn et al. 2012 (102)	Emotional CPT	10	10	0.40	14.3
ANX	Yin et al. 2017 (103)	Valence Evaluation Task	14	20	0.25	15.7
ANX	Burkhouse et al. 2018 (104)	Emotional Conflict Task	25	25	0.41	15.3
ANX	Blair et al. 2011 (105)	Emotional Face Task	16	14	0.50	13.3
DEP	Chantiluke et al. 2012_1 (106)	Reward Continuous Performance	21	20	0.50	16.2
DEP	Chantiluke et al. 2012_2 (106)	Reward Continuous Performance	21	20	0.50	16.2
DEP	Colich et al. 2015 (107)	modified affective Go/No-Go	15	18	0.17	15.6
DEP	Davey et al. 2011 (108)	Being-Liked (Emotional Face)	20	19	0.35	18.6
DEP	Diler et al. 2013 (109)	emotional faces (gender)	10	10	0.20	15.9
DEP	Diler et al. 2014 (110)	Go/No-Go	10	10	0.20	15.9
DEP	Gaffrey et al. 2013 (111)	Facial Emotion Viewing Task	31	23	0.57	5.0
DEP	Halari et al. 2009_1 (112)	Simon Task	21	21	0.48	16.2
DEP	Halari et al. 2009_2 (112)	Switch Task	21	21	0.48	16.2
DEP	Halari et al. 2009_3 (112)	Stop Task	21	21	0.48	16.2
DEP	Hall et al. 2014 (113)	Emotional Faces Task	23	32	0.26	15.5
DEP	Roberson-Nay et al. 2006 (114)	Emotional Face Task: Encoding	23	10	0.30	13.8
DEP	Sharp et al. 2014 (115)	Modified card-guessing game	19	14	0.00	13.4
DEP	Tao et al. 2012 (116)	Emotional Face Task (gender)	21	19	0.42	14.2
DEP	Yang et al. 2009 (117)	Stop Task	13	13	0.46	16.0
DEP	Yang et al. 2010 (118)	facial-emotion matching task	12	12	0.58	15.9
DEP	Pan et al. 2011 (119)	Go/No-Go task	-	15	0.47	15.9
DEP	Pan et al. 2013 (A) (120)	Iowa Gambling Task	13	15	0.27	16.2
DEP	Pan et al. 2013 (B) (120)	Iowa Gambling Task	-	14	0.50	15.8
DEP	Groschwitz et al. 2016 (121)	Cyberball Task	15	14	0.21	15.4
DEP	Suzuki et al. 2014 (A) (122)	Emotional Face Task	51	42	0.48	9.8
DEP	Suzuki et al. 2014 (B) (122)	Emotional Face Task	-	22	0.41	10.2
DEP	De Bellis et Hooper 2013 (123)	Emotional oddball task	5	5	0.40	15.5

Note. (A)(B) = distinct samples from the same study. $_{1\text{-}2}$ = distinct task contrasts or fMRI task from the same study. MED = Medication (in %); COG = Cognitive; RI = Response Inhibition; ATTN = Attention; EMO = Emotional; BOTH = Both Positive and Negative Emotion; POS = Positive; NEG = Negative;

Supplementary Table 2. Included studies: Medication and task-characteristics

Primary Diagnosis	First Author, Year	MED (%)	Task-Characteristics							
			COG	RI	ATTN	EMO	BOTH	POS	NEG	Others
ADHD	Braet et al. 2011 (1)	NA	X	X						
ADHD	Bédard et al. 2014 (2)	0.08	X							
ADHD	Bollmann et al. 2017 (3)	0.67	X							
ADHD	Booth et al. 2005 (4)	1.00	X	X						
ADHD	Cao et al. 2008 (5)	0.25	X		X					
ADHD	Cerullo et al. 2009 (6)	0.00	X		X					
ADHD	Chou et al. 2015_1 (7)	0.00	X	X						
ADHD	Chou et al. 2015_2 (7)	0.00	X	X						
ADHD	Christakou et al. 2013 (8)	0.00	X		X					
ADHD	Durston et al. 2006 (9)	0.55	X	X						
ADHD	Durston et al. 2007_1 (10)	0.75	X	X						
ADHD	Durston et al. 2007_2 (10)	0.75	X	X						
ADHD	Ercan et al. 2016 (11)	NA	X	X						
ADHD	Fan et al. 2014 (12)	0.24	X	X						
ADHD	Hart et al. 2014 (13)	0.30	X	X						
ADHD	Hart et al. 2014 (14)	0.30	X	X						
ADHD	Hauser et al. 2014 (15)	0.80				X		X		
ADHD	Hwang et al. 2015 (16)	0.42	X	X		X	X			
ADHD	Iannaccone et al. 2015_1 (17)	0.72	X	X						
ADHD	Iannaccone et al. 2015_2 (17)	0.72	X	X						
ADHD	Janssen et al. 2015 (18)	0.90	X	X						
ADHD	Kohls et al. 2014 (19)	0.56				X		X		
ADHD	Konrad et al. 2006 (20)	0.00	X		X					
ADHD	Krauel et al. 2007 (21)	0.42	X							
ADHD	Li et al. 2014 (22)	0.00	X							
ADHD	Ma et al. 2012 (23)	0.20	X	X						
ADHD	Ma et al. 2016 (24)	0.60				X		X		
ADHD	Massat et al. 2012 (25)	0.00	X							
ADHD	Massat et al. 2018 (26)	0.00	X	X						
ADHD	Metin et al. 2017 (27)	0.00				X		X		
ADHD	Mostofsky et al. 2006 (28)	0.73								X
ADHD	Norman et al. 2017 (29)	0.35	X		X					
ADHD	Norman et al. 2018 (30)	0.50				X	X			
ADHD	O'Conaill et al. 2015 (31)	0.79	X		X					
ADHD	Orinstein et al. 2014 (32)	NA	X		X					
ADHD	Passarotti et al. 2010 (33)	0.00				X	X			
ADHD	Passarotti et al. 2010 (34)	0.00				X	X			
ADHD	Passarotti et al. 2010 (35)	0.00	X	X						
ADHD	Poissant et al. 2016 (A) (36)	NA	X							
ADHD	Poissant et al. 2016 (B) (36)	NA	X							
ADHD	Posner et al. 2011 (37)	0.00				X			X	
ADHD	Posner et al. 2011 (38)	0.53				X	X			
ADHD	Rubia et al. 2005 (39)	0.00	X	X						
ADHD	Rubia et al. 2008 (40)	0.00	X	X						
ADHD	Rubia et al. 2009 (41)	0.00	X		X	X		X		
ADHD	Rubia et al. 2009 (42)	0.00	X	X						
ADHD	Rubia et al. 2009 (43)	0.00				X		X		
ADHD	Rubia et al. 2010 (44)	0.00	X		X					

ADHD	Rubia et al. 2010_1 (45)	0.00	X	X						
ADHD	Rubia et al. 2010_2 (45)	0.00	X		X					
ADHD	Rubia et al. 2011 (46)	0.00	X	X						
ADHD	Schwarz et al. 2015 (47)	0.91								X
ADHD	Sheridan et al. 2007 (48)	0.20	X							
ADHD	Silk et al. 2008 (49)	0.00	X							
ADHD	Siniatchkin et al. 2012 (50)	0.75	X	X						
ADHD	Smith et al. 2008 (51)	0.00	X							
ADHD	Spinelli et al. 2011 (52)	0.15	X	X						
ADHD	Spinelli et al. 2011 (53)	0.15	X	X						
ADHD	Stevens et al. 2017_1 (A) (54)	0.68	X							
ADHD	Stevens et al. 2017_2 (A) (54)	0.68				X		X		
ADHD	Stevens et al. 2017_1 (B) (54)	0.77	X	X						
ADHD	Stevens et al. 2017_2 (B) (54)	0.77				X		X		
ADHD	Stevens et al. 2017 (C) (54)	0.67	X							
ADHD	Tamm et al. 2004 (55)	0.50	X	X						
ADHD	Tamm et al. 2006 (56)	0.36	X		X					
ADHD	Tamm et al. 2012 (57)	0.00	X							
ADHD	Tegelbeckers et al. 2015 (58)	0.53	X		X					
ADHD	Vance et al. 2007 (59)	0.00	X							
ADHD	van Ewijk et al. 2015 (60)	0.83	X							
ADHD	van Rooij et al. 2015 (61)	0.54	X	X						
ADHD	Vetter et al. 2018 (62)	NA	X			X			X	
ADHD	Vloet et al. 2010 (63)	NA	X		X					
ADHD	von Rhein et al. 2015 (64)	0.76				X		X		
ADHD	Wang et al. 2013 (65)	0.00	X		X					
ADHD	Hegarty et al. 2021 (66)	0.00	X	X						
ADHD	Finger et al., 2008 (67)	0.71				X	X			
ADHD	Gatzke-Kopp et al. 2009 (68)	0.53				X		X		
ADHD	Bubenzer-Busch et al. 2016_1 (69)	0.85				X			X	
ADHD	Bubenzer-Busch et al. 2016_2 (69)	0.85				X		X		
CD/ODD	Banich et al. 2007 (70)	NA	X	X						
CD/ODD	Bjork et al. 2010 (71)	0.67				X		X		
CD/ODD	Crowley et al. 2010_1 (72)	0.30				X		X		
CD/ODD	Crowley et al. 2010_2 (72)	0.30				X			X	
CD/ODD	Dong et al. 2017 (73)	NA				X			X	
CD/ODD	Fairchild et al. 2014 (74)	NA				X			X	
CD/ODD	Fehlbaum et al. 2018 (75)	0.90	X	X		X			X	
CD/ODD	Finger et al. 2011_1 (76)	0.47				X		X		
CD/ODD	Finger et al. 2011_2 (76)	0.47				X			X	
CD/ODD	Herpertz et al. 2008 (77)	NA				X			X	
CD/ODD	Hwang et al. 2016 (A) (78)	0.24	X	X		X			X	
CD/ODD	Hwang et al. 2016 (B) (78)	0.28	X	X		X			X	
CD/ODD	Klapwijk et al., 2016a (79)	0.00				X			X	
CD/ODD	Klapwijk et al., 2016b (80)	0.00	X							
CD/ODD	Marsh et al., 2011 (81)	0.43	X							
CD/ODD	Marsh et al., 2013 (82)	0.14	X			X			X	
CD/ODD	Passamonti et al., 2010 (A) (83)	NA				X			X	
CD/ODD	Passamonti et al., 2010 (B) (83)	NA				X			X	
CD/ODD	Rubia et al. 2008 (40)	0.00	X	X						
CD/ODD	Rubia et al. 2009 (41)	0.00	X		X	X	X			
CD/ODD	Rubia et al. 2009 (42)	0.00	X	X						

CD/ODD	Rubia et al. 2010 (44)	0.00	X		X				
CD/ODD	Schwenck et al., 2017 (84)	0.33				X			X
CD/ODD	Thornton et al., 2017 (85)	0.14	X		X				
CD/ODD	White et al., 2012a (86)	0.12	X		X	X			X
CD/ODD	White et al., 2012b (87)	0.12				X			X
CD/ODD	White et al., 2013$_1$ (88)	0.10				X		X	
CD/ODD	White et al., 2013$_2$ (88)	0.10				X			X
CD/ODD	White et al., 2014 (89)	0.20				X	X		
CD/ODD	Zhang et al., 2015 (90)	0.00	X	X					
CD/ODD	Zhu et al. 2014 (91)	0.00	X	X					
CD/ODD	Raschle et al. 2019 (92)	NA				X			X
ANX	Yang et al. 2004 (93)	0.00				X			X
ANX	Carrion et al. 2008 (94)	0.00	X	X					
ANX	Keding-Herringa et al. 2016 (95)	0.00				X	X		
ANX	Hart et al. 2018 (96)	0.00				X			X
ANX	Carlisi et al. 2017 (97)	0.00				X			X
ANX	Gold et al. 2020 (98)	0.00				X			X
ANX	Thomas et al. 2001 (99)	0.00				X			X
ANX	Monk et al. 2006 (100)	0.00				X			X
ANX	Monk et al. 2008 (101)	0.00				X			X
ANX	Strawn et al. 2012 (102)	0.00	X		X	X	X		
ANX	Yin et al. 2017 (103)	0.00				X			X
ANX	Burkhouse et al. 2018 (104)	0.00				X			X
ANX	Blair et al. 2011 (105)	0.00				X			X
DEP	Chantiluke et al. 2012$_1$ (106)	0.00	X		X	X		X	
DEP	Chantiluke et al. 2012$_2$ (106)	0.00	X		X	X		X	
DEP	Colich et al. 2015 (107)	0.39	X	X		X			X
DEP	Davey et al. 2011 (108)	0.47				X			
DEP	Diler et al. 2013 (109)	0.60				X	X		
DEP	Diler et al. 2014 (110)	0.47	X	X					
DEP	Gaffrey et al. 2013 (111)	0.00				X	X		
DEP	Halari et al. 2009$_1$ (112)	0.00	X	X					
DEP	Halari et al. 2009$_2$ (112)	0.00	X		X				
DEP	Halari et al. 2009$_3$ (112)	0.00	X	X					
DEP	Hall et al. 2014 (113)	0.00				X			X
DEP	Roberson-Nay et al. 2006 (114)	0.00	X			X	X		
DEP	Sharp et al. 2014 (115)	0.00				X		X	
DEP	Tao et al. 2012 (116)	0.00				X			X
DEP	Yang et al. 2009 (117)	0.00	X	X					
DEP	Yang et al. 2010 (118)	0.00				X	X		
DEP	Pan et al. 2011 (119)	0.47	X	X					
DEP	Pan et al. 2013 (A) (120)	0.67				X	X		
DEP	Pan et al. 2013 (B) (120)	0.43				X	X		
DEP	Groschwitz et al. 2016 (121)	0.36				X			X
DEP	Suzuki et al. 2014 (A) (122)	NA				X			X
DEP	Suzuki et al. 2014 (B) (122)	NA				X			X
DEP	De Bellis et Hooper 2013 (123)	0.00	X		X	X			X

Note. (A)(B) = distinct samples from the same study. $_{1\text{-}2}$ = distinct task contrasts or fMRI task from the same study. MED = Medication (in %); COG = Cognitive; RI = Response Inhibition; ATTN = Attention; EMO = Emotional; BOTH = Both Positive and Negative Emotion; POS = Positive; NEG = Negative;

Additional information about the Correlation-Matrix Hierarchical Clustering.

We thus repeated the CMHC for a range of 2 to 15 MAGs. The optimal number of MAGs was determined by comparing frequently used metrics in fMRI clustering (i.e., silhouette and calinski-harabasz indices, variation of information & adjusted rand index) (55), for each of the 2-15 MAGs (See Supplementary Material for more information). More precisely, we used a 90% subsampling resample strategy (without replacement, 5000 iterations). This implied that we randomly removed 10% of experiments, iteratively, to perturbate the ability to group the most correlated experiments with each other, successively. Then, for K range, each metric was compared against a null distribution. To do so, 5,000 datasets were created artificially by shuffling foci locations across experiments but preserving original experiments' properties (e.g., number of foci, sample size). The average of each metric (i.e., silhouette and calinski-harabasz indices) derived from *true* dataset, was normalized using this null distribution ($Average_{TRUE} – Average_{NULL} / STD_{NULL}$) and then plotted for K range (2-15 MAGs). This improves our ability to select the optimal K by taking into account the probabilities of getting a certain metric value in a random spatial arrangement. Given that the ground truth class labels are unknown, we compared, for each K, the consistency (adjusted rand index) and shared information distance (variation of information) between $Label_{TRUE}$ with the $Label_{NULL}$ then averaged across the 5000 iterations. These metrics were then plotted for K range. A local minimum in the plot suggests a decrease in overlap between both sets of labels. After finding the optimal number of MAGs, the most stable label solution was found by grouping experiments that were labelled similarly across the 5,000 subsampling iterations. More precisely, we calculated the hamming distance between each experiment's label ids (147 experiments by 5000 iterations ids) to calculate the proportion of disagreement between two experiments' set of labels. A CMHC was performed on a final 147-by-147 experiments'

distance matrix, representing the distance between each pair of experiments. We then used the determined most optimal number of MAGs to extract the most stable label ids.

Supplementary Figure 1. Metrics computed for K=2 to 15 clustering solutions. **A.** This line graph represents silhouette (Rousseeuw, 1986) and calinski-harabasz (Calinski et Harabasz, 1974) metrics averaged over 5,000 subsampling iterations and then normalized against a null distribution. Green line = Silhouette metric, Blue line = Calinski-Harabasz metric. **B.** Line graph refers to changes in silhouette and calinski-harabasz values from K to K+1 (i.e. (K+1) – 1). Silhouette metrics (green) show a stable solution at k=8, while calinski-harabasz metric was monotonic. **C.** Adjusted Rand Indices between K_{TRUE} - K_{NULL}. This plot shows lowest scores at K=3, K=8. **D.** Variation of Information between K_{TRUE} - K_{NULL}. Although scores are rather monotonic, greatest increases moving from K=2 to 3, K=6 to K=7 and K=7 to K=8.

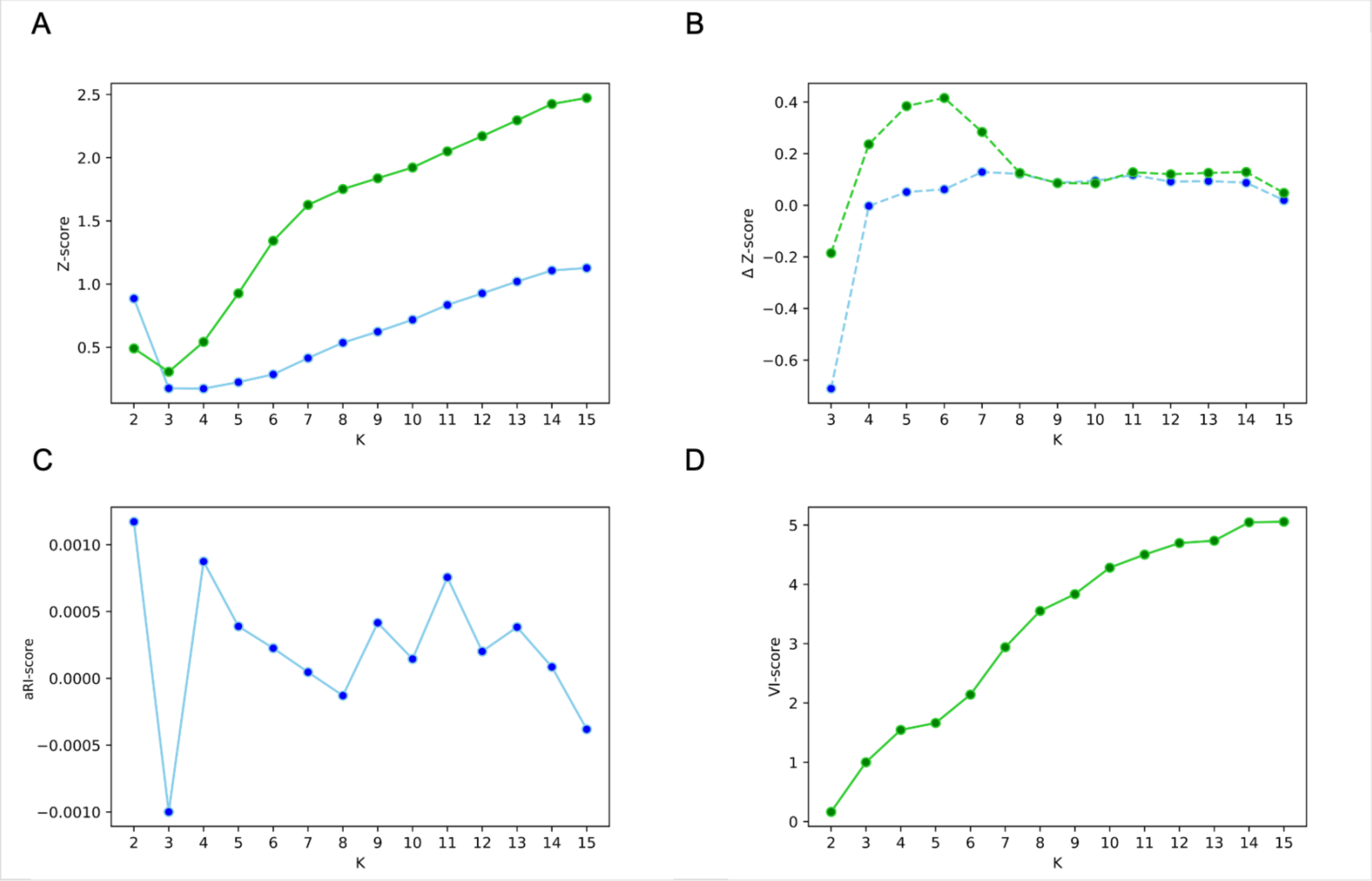

Supplementary Figure 2. Z-scores of probabilities of aberrant MAG per disorder class. For each disorder class, z-score was calculated by subtracting the average voxelwise ALE probability (whole brain) from the average ALE probability within MAG divided by the standard deviation of the whole-brain ALE probabilities.

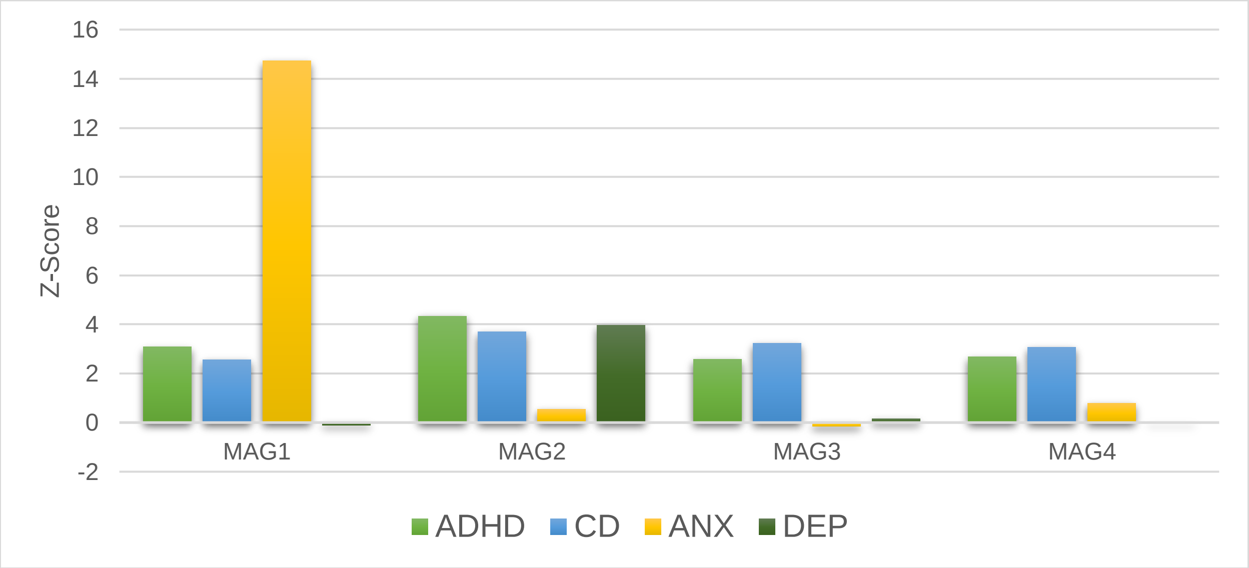

Supplementary Figure 3. Results from the forward (classical) ALE meta-analysis. Red cluster = Transdiagnostic Neurobiological Marker when pooling ADHD, CD/ODD, ANX and ADHD (x=4, y=24, z=42, ALE score=0.021, 323 voxels). Green Cluster = Externalizing Disorders (x=4, y=26, z=44, maximum ALE score=0.0279, 147 voxels); Blue Cluster = Internalizing Disorders (x=-8, y=38, z=8, maximum ALE score = 0.0167, 129 voxels).

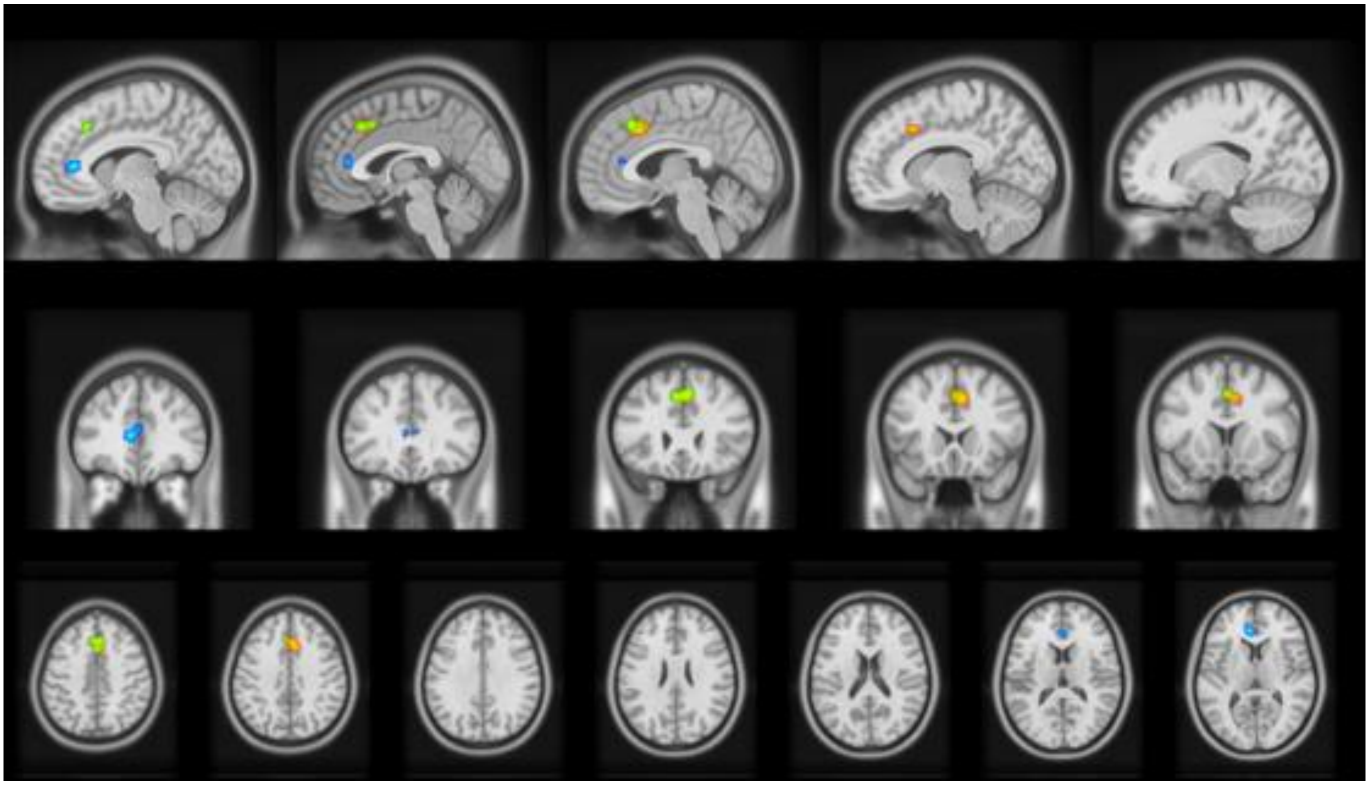

Supplementary Table 3. Results from classical meta-analysis on nosological categories

Diagnosis Category	MNI Coordinates			ALE	Cluster Size
	X	Y	Z		
ADHD					
dACC	-2	12	22	0.0198	51
vlPFC (BA47)	50	14	-4	0.0179	35
CD/ODD					
Caudate	12	8	0	0.0118	40
pre-SMA	-6	26	42	0.0113	28
ANX					
N.S.	-	-	-	-	-
DEP					
N.S.	-	-	-	-	-

Note. These results were performed using liberal threshold (p=0.001 uncorrected at a voxel-level). ADHD = Attention deficit hyperactivity disorder; CD/ODD = Conduct Disorder / Oppositional Defiant Disorder; ANX = Anxiety Disorder; DEP = Depressive Disorder. MNI = Montreal Neurological Institute; N.S. = Not Statistically Significant. dACC = dorsal Anterior Cingulate Cortex; vlPFC = ventrolateral Prefrontal Cortex; pre-SMA = pre-Supplementary Motor Area.

Supplementary Table 4. List of fMRI tasks per MAGs.

MAG1
N-Back
Go-No/Go
Go-No/Go
Reversal Learning Task
Rewarded Go-No/Go
Rewarded Stroop Task
Response Inhibition Task
Stop Task
Simon Task
Go-No/Go
Spatial span task
monetary incentive delay task
Emotional face probe detection task
Continuous Performance Task with emotional distractors
face-attention paradigm
Colorado Balloon Game
Colorado Balloon Game
Probabilistic Reversal Task
Monetary Incentive task
Simon Task
Probabilistic Reversal Task

MAG2
Go-No/Go
Spatial Working Memory Task
Go-No/Go
Cued Target Detection
Stroop Task
Continuous Performance Task
Sustained Attention Task
Go-No/Go
Stroop Task
Stop Task
Speeded Flanker Task
Speeded Flanker Task
Stop-Signal Task
Attention Network Test
Recognition Test
N-Back
N-Back
Spatial Attention Paradigm
Sustained Attention Task
Iowa Gambling Task
Visual Search Task
Auditory Oddball Attention Task
N-Back
Emotional Valence Stroop Task
Subliminal Presentation of Fearful Faces
Emotional Stroop Task
Visual–Spatial Switch Task
Stop Task
Switch Task
Raven's Progressive Matrices Task
Go/NoGo
Time Discrimination
Monetary incentive delay task
Monetary incentive delay task
Monetary incentive delay task

monetary incentive delay task
Go-No/Go
Stop-Signal Task
Continuous Performance Task
Passive-Viewing Emotional Face Task
Emotional Face visual probe task
Valence Evaluation Task
Emotional Conflict Task
Emotional Face Task
Reward Continuous Performance Task
modified affective Go/No-Go
Being-Liked Task (Emotional Face)
emotional facial expression gender labeling task
Go/No-Go
Simon Task
Stop Task
Emotional Faces Task
Emotional Face Task: Encoding
Modified card-guessing game (monetary reward)
Emotional Face Task (gender labelling)
Parametric inhibitory task (Stop task)
facial-emotion matching task
Go/No-Go task
Iowa Gambling Task
Iowa Gambling Task
Emotional Perception
Go-No/Go
Dynamic Face Task
Emotional oddball task
Emotional oddball task
Cyberball Task
Stop-Signal Task
GoStop Task
Stroop Task
Monetary Incentive Delay (MID) task
Point Subtraction Aggression Game
Point Subtraction Aggression Game
Affective Stroop Task
Passive-Viewing Emotional Stimuli Task
Affective Stroop Task
Affective Stroop Task
Empathic Emotional Face Task
Dictator Game
Empathic Situation of Pain Task
Emotional Face Task
Emotional Face Task
Monetary gambling task
Animacy Attention Task
Passive Avoidance Task
Passive Avoidance Task
GoStop Task
Stroop Task

MAG3

N-Back
Finger Sequencing Paradigm
Stop Task
Simon Task
Anti-Saccade and Fixation Trials
Match-to-Sample Task
Oddball Task
Mental Rotation Task

Facial Emotion Viewing Task
Stop Task
Visual–Spatial Switch Task
Eye Gaze Task
Doors Task

MAG4

Go-No/Go
Stop Task
Stroop Paradigm
Go-No/Go
Pictures Story
Reward Continuous Performance Task
Fluid Reasoning Task
Visual Oddball Task
Threat conditioning
Emotional Face Task
Implicit Association Test (words)
Rewarded Continuous Performance Test (CPT)

Supplementary Phenotype Assessment: Literature Bias & Subanalyses

We tested if fMRI literature showed differences between nosological categories regarding the choice of neurocognitive task domains, average of sex ratio, and the average of prescribed medication per samples. Literature showed significant differences in cognitive tasks (X_2=23.75, p<0.001), general emotional tasks (X_2=42.11, p<0.001) but more specifically in tasks using simultaneously positive and negative stimuli (X_2=7.85, p=0.049) and negative stimuli only (X_2=53.69, p<0.001). Biases were also observed in the average of sex ratio (X_2=64.69, p<0.001) and the average of sample with prescribed medication (X_2=18.02, p<0.001).

Given that these factors may alter the relationships between nosological categories and MAGs, further analyses were performed by restricting experiments using specific cognitive task contrasts, emotional task contrasts, medication naïve and mixed sex samples. Subanalyses on emotion-specific stimuli (e.g., positive, negative or both) could not be performed due to the limited sample size across MAGs. First, the lower rates of DEP samples across MAG1 compared to other MAGs was only replicated when restricting experiments to those using an emotional task contrast (X_2=4.34, p=0.037) and a mixed sex samples (X_2=3.89, p=0.049). Furthermore, MAG1 had higher rates of ADHD samples in experiments with an emotional task contrast (X_2=3.18, p=0.050).

Finally, the relationship between MAG2 and DEP samples was replicated when restricting experiments to those using a cognitive task contrast (X_2=5.53, p=0.019), an emotional task contrast (X_2=5.22, p=0.022), but also in experiments with only medication naïve sample (X_2=5.70, p=0.017) and in mixed sex samples (X_2=7.54, p=0.006).

Supplementary Table 5. Disorder-specific sample and task characteristics

Characteristics	ADHD	CD/ODD	ANX	DEP
Task-domain				
Cognitive	60 (75.9%)	15 (46.9%)	2 (14.3%)	11 (50.0%)
RI	29 (36.7%)	8 (25%)	1 (7.1%)	6 (27.3%)
Attention	14 (17.7%)	4 (12.5%)	1 (7.1%)	4 (18.2%0
Emotion	19 (24.1%)	23 (71.9%)	13 (92.9%)	16 (72.7%)
Positive	10 (12.7%)	4 (12.5%)	0 (0%)	3 (13.6%)
Negative	3 (3.8%)	17 (53.1%)	11 (78.6%)	6 (27.3%)
Both	6 (7.6%)	2 (6.3%)	2 (14.3%)	6 (27.3%)
Sample				
Average Med per sample (Mean, SD)	35.5 (33.9)	21.5 (23.7)	0	19.26 (25.0)
Average Boys per Sample (Mean, SD)	82.2 (21.4)	81.4 (27.3)	44.4 (14.7)	37.5 (15.2)

Note. RI = Response Inhibition; Both = Both Positive and Negative Emotional Stimuli; Med = Medication

Supplementary Table 6. Disorder-specific comorbidities

	Comorbidities							
	ADHD		CD/ODD		ANX		DEP	
Primary Diagnosis	n	mean %	n	mean %	n	mean %	n	mean %
ADHD (n=79)	-	-	65	14.4 (16.3)	64	1.7 (4.6)	65	1.2 (4.7)
CD/ODD (n=32)	29	33,4 (24.1)	-	-	31	1.0 (3.1)	31	5.9 (19.6)
ANX (n=14)	6	10.5 (15.6)	10	6.7 (14.9)	-	-	12	12 (28.2)
DEP (n=22)	20	7.2 (9.0)	20	4.1 (8.8)	21	23.6 (27.1)	-	-

Note. *n* = the number of samples with available data.

Supplementary Table 7. Disorder-specific comorbidities per MAGs

Characteristics	MAG1 (k=21)		MAG2 (k=87)		MAG3 (k=13)		MAG4 (k=12)	
	n	%	n	%	n	%	n	%
ADHD samples								
ANX	12	3.33%	32	1.53%	8	1.13%	7	1.14%
DEP	11	3.46%	34	0.65%	8	2.25%	7	0%
CD/ODD	12	23.58%	33	15.50%	8	6.63%	7	5.14%
ANX samples								
ADHD	0	0%	4	4.50%	0	0%	1	4%
DEP	2	46.50%	8	18.13%	0	0%	1	0%
CD/ODD	2	0%	6	2.50%	0	0%	1	4%
**DEP samples**								
ADHD	0	0%	16	6.75%	1	13%	0	0%
ANX	0	0%	18	24.50%	1	30%	0	0%
CD/ODD	0	0%	16	2.06%	1	17%	0	0%
**CD/ODD samples**								
ADHD	2	33.50%	16	36.19%	4	25%	3	26.30%
ANX	4	0%	16	1.25%	4	0%	3	1.67%
DEP	4	0%	16	1.69%	4	0%	3	15%

Note. n = the number of samples with available data. The only analyses were performed in samples with ADHD and revealed marginally significant differences between MAGs relatively to comorbidities with CD/ODD (H=7.11, p=0.068). Post-hoc tests suggested that in ADHD samples, rate of CD/ODD comorbidity was higher is MAG1 compared to MAG3 (p=0.012) and MAG4 (p=0.011) but not compared to MAG2 (p=0.142). No differences were observed for ANX or DEP comorbidities in ADHD samples

Disorder-Specific ALE Meta-analyses

Meta-analyses were performed across all foci, independently of the activation direction, for ANX (46 foci, 14 experiments, 293 subjects), DEP (103 foci, 22 experiments, 388 subjects), ADHD (700 foci, 79 experiments, 1892 subjects) and CD (197 foci, 32 experiments, 626 subjects). However, each disorder-specific meta-analysis revealed no significant spatial convergence (See Supplementary Material for results using uncorrected threshold). Nonetheless, we observed peak convergence in dorsal/perigenual ACC (MNI x=-8, y=38, z=8, maximum ALE score = 0.0167, 129 voxels) for the internalizing category (ANX+DEP) and pre-SMA (x=4, y=26, z=44, maximum ALE score=0.0279, 147 voxels) for the externalizing category (ADHD+CD/ODD). Additionally, merging experiments across pediatric psychiatric disorders, we observed a significant cluster that included voxels of the right anterior MCC and the pre-supplementary motor area (x=4, y=24, z=42, ALE score=0.021, 323 voxels). Despite the fact that ADHD (57.14%) and CD/ODD (35.71%) largely contributed to this specific brain region, proportions did not significantly differ from their respective base rate. Furthermore, no significant differences were observed regarding nosological categories nor task-characteristics. However, Mann-Whitney U revealed a significant sex effect, indicating that the aMCC/pre-SMA may be associated with higher average rates of boys across samples (M=69.7% versus M=91.77%; U=479.0; p=0.003). No other significant differences were observed.

REFERENCES

1. Braet W, Johnson KA, Tobin CT, Acheson R, McDonnell C, Hawi Z, et al. (2011): fMRI activation during response inhibition and error processing: the role of the DAT1 gene in typically developing adolescents and those diagnosed with ADHD. *Neuropsychologia*. 49:1641-1650.
2. Bédard A-CV, Newcorn JH, Clerkin SM, Krone B, Fan J, Halperin JM, et al. (2014): Reduced prefrontal efficiency for visuospatial working memory in attention-deficit/hyperactivity disorder. *Journal of the American Academy of Child & Adolescent Psychiatry*. 53:1020-1030. e1026.
3. Bollmann S, Ghisleni C, Poil S-S, Martin E, Ball J, Eich-Höchli D, et al. (2017): Age-dependent and-independent changes in attention-deficit/hyperactivity disorder (ADHD) during spatial working memory performance. *The World Journal of Biological Psychiatry*. 18:279-290.
4. Booth JR, Burman DD, Meyer JR, Lei Z, Trommer BL, Davenport ND, et al. (2005): Larger deficits in brain networks for response inhibition than for visual selective attention in attention deficit hyperactivity disorder (ADHD). *Journal of Child Psychology and Psychiatry*. 46:94-111.
5. Cao Q, Zang Y, Zhu C, Cao X, Sun L, Zhou X, et al. (2008): Alerting deficits in children with attention deficit/hyperactivity disorder: event-related fMRI evidence. *Brain research*. 1219:159-168.
6. Cerullo MA, Adler CM, Lamy M, Eliassen JC, Fleck DE, Strakowski SM, et al. (2009): Differential brain activation during response inhibition in bipolar and attention-deficit hyperactivity disorders. *Early intervention in psychiatry*. 3:189-197.
7. Chou T-L, Chia S, Shang C-Y, Gau SS-F (2015): Differential therapeutic effects of 12-week treatment of atomoxetine and methylphenidate on drug-naive children with attention deficit/hyperactivity disorder: A counting Stroop functional MRI study. *European Neuropsychopharmacology*. 25:2300-2310.
8. Christakou A, Murphy C, Chantiluke K, Cubillo A, Smith A, Giampietro V, et al. (2013): Disorder-specific functional abnormalities during sustained attention in youth with attention deficit hyperactivity disorder (ADHD) and with autism. *Molecular psychiatry*. 18:236-244.
9. Durston S, Mulder M, Casey B, Ziermans T, van Engeland H (2006): Activation in ventral prefrontal cortex is sensitive to genetic vulnerability for attention-deficit hyperactivity disorder. *Biological psychiatry*. 60:1062-1070.
10. Durston S, Davidson MC, Mulder MJ, Spicer JA, Galvan A, Tottenham N, et al. (2007): Neural and behavioral correlates of expectancy violations in attention-deficit hyperactivity disorder. *Journal of Child Psychology and Psychiatry*. 48:881-889.
11. Ercan ES, Suren S, Bacanlı A, Yazici KU, Callı C, Ozyurt O, et al. (2016): Decreasing ADHD phenotypic heterogeneity: searching for neurobiological underpinnings of the restrictive inattentive phenotype. *European child & adolescent psychiatry*. 25:273-282.
12. Fan L, Gau SS, Chou T (2014): Neural correlates of inhibitory control and visual processing in youths with attention deficit hyperactivity disorder: a counting Stroop functional MRI study. *Psychological Medicine*. 44:2661.
13. Hart H, Marquand AF, Smith A, Cubillo A, Simmons A, Brammer M, et al. (2014): Predictive neurofunctional markers of attention-deficit/hyperactivity disorder based on pattern classification of temporal processing. *Journal of the American Academy of Child & Adolescent Psychiatry*. 53:569-578. e561.

14. Hart H, Chantiluke K, Cubillo AI, Smith AB, Simmons A, Brammer MJ, et al. (2014): Pattern classification of response inhibition in ADHD: toward the development of neurobiological markers for ADHD. *Human Brain Mapping*. 35:3083-3094.
15. Hauser TU, Iannaccone R, Ball J, Mathys C, Brandeis D, Walitza S, et al. (2014): Role of the medial prefrontal cortex in impaired decision making in juvenile attention-deficit/hyperactivity disorder. *JAMA psychiatry*. 71:1165-1173.
16. Hwang S, White SF, Nolan ZT, Williams WC, Sinclair S, Blair R (2015): Executive attention control and emotional responding in attention-deficit/hyperactivity disorder—A functional MRI study. *NeuroImage: Clinical*. 9:545-554.
17. Iannaccone R, Hauser TU, Ball J, Brandeis D, Walitza S, Brem S (2015): Classifying adolescent attention-deficit/hyperactivity disorder (ADHD) based on functional and structural imaging. *European child & adolescent psychiatry*. 24:1279-1289.
18. Janssen TW, Heslenfeld DJ, van Mourik R, Logan GD, Oosterlaan J (2015): Neural correlates of response inhibition in children with attention-deficit/hyperactivity disorder: A controlled version of the stop-signal task. *Psychiatry Research: Neuroimaging*. 233:278-284.
19. Kohls G, Thönessen H, Bartley GK, Grossheinrich N, Fink GR, Herpertz-Dahlmann B, et al. (2014): Differentiating neural reward responsiveness in autism versus ADHD. *Developmental cognitive neuroscience*. 10:104-116.
20. Konrad K, Neufang S, Hanisch C, Fink GR, Herpertz-Dahlmann B (2006): Dysfunctional attentional networks in children with attention deficit/hyperactivity disorder: evidence from an event-related functional magnetic resonance imaging study. *Biological psychiatry*. 59:643-651.
21. Krauel K, Duzel E, Hinrichs H, Santel S, Rellum T, Baving L (2007): Impact of emotional salience on episodic memory in attention-deficit/hyperactivity disorder: a functional magnetic resonance imaging study. *Biological Psychiatry*. 61:1370-1379.
22. Li Y, Li F, He N, Guo L, Huang X, Lui S, et al. (2014): Neural hyperactivity related to working memory in drug-naive boys with attention deficit hyperactivity disorder. *Progress in Neuro-Psychopharmacology and Biological Psychiatry*. 53:116-122.
23. Ma J, Lei D, Jin X, Du X, Jiang F, Li F, et al. (2012): Compensatory brain activation in children with attention deficit/hyperactivity disorder during a simplified Go/No-go task. *Journal of neural transmission*. 119:613-619.
24. Ma I, van Holstein M, Mies GW, Mennes M, Buitelaar J, Cools R, et al. (2016): Ventral striatal hyperconnectivity during rewarded interference control in adolescents with ADHD. *Cortex*. 82:225-236.
25. Massat I, Slama H, Kavec M, Linotte S, Mary A, Baleriaux D, et al. (2012): Working memory-related functional brain patterns in never medicated children with ADHD. *PloS one*. 7:e49392.
26. Massat I, Slama H, Villemonteix T, Mary A, Baijot S, Albajara Sáenz A, et al. (2018): Hyperactivity in motor response inhibition networks in unmedicated children with attention deficit-hyperactivity disorder. *The World Journal of Biological Psychiatry*. 19:101-111.
27. Metin B, Tas ZC, Çebi M, Büyükaslan A, Soysal A, Hatıloglu D, et al. (2018): Reward processing deficits during a spatial attention task in patients with ADHD: an fMRI study. *Journal of attention disorders*. 22:694-702.
28. Mostofsky SH, Rimrodt SL, Schafer JG, Boyce A, Goldberg MC, Pekar JJ, et al. (2006): Atypical motor and sensory cortex activation in attention-deficit/hyperactivity disorder: a functional magnetic resonance imaging study of simple sequential finger tapping. *Biological psychiatry*. 59:48-56.

29. Norman LJ, Carlisi CO, Christakou A, Cubillo A, Murphy CM, Chantiluke K, et al. (2017): Shared and disorder-specific task-positive and default mode network dysfunctions during sustained attention in paediatric Attention-Deficit/Hyperactivity Disorder and obsessive/compulsive disorder. *NeuroImage: Clinical*. 15:181-193.
30. Norman LJ, Carlisi CO, Christakou A, Murphy CM, Chantiluke K, Giampietro V, et al. (2018): Frontostriatal dysfunction during decision making in attention-deficit/hyperactivity disorder and obsessive-compulsive disorder. *Biological Psychiatry: Cognitive Neuroscience and Neuroimaging*. 3:694-703.
31. O'Conaill CR, Malisza KL, Buss JL, Bolster RB, Clancy C, de Gervai PD, et al. (2015): Visual search for feature conjunctions: an fMRI study comparing alcohol-related neurodevelopmental disorder (ARND) to ADHD. *Journal of neurodevelopmental disorders*. 7:1-18.
32. Orinstein AJ, Stevens MC (2014): Brain activity in predominantly-inattentive subtype attention-deficit/hyperactivity disorder during an auditory oddball attention task. *Psychiatry Research: Neuroimaging*. 223:121-128.
33. Passarotti AM, Sweeney JA, Pavuluri MN (2010): Emotion processing influences working memory circuits in pediatric bipolar disorder and attention-deficit/hyperactivity disorder. *Journal of the American Academy of Child & Adolescent Psychiatry*. 49:1064-1080.
34. Passarotti AM, Sweeney JA, Pavuluri MN (2010): Differential engagement of cognitive and affective neural systems in pediatric bipolar disorder and attention deficit hyperactivity disorder. *Journal of the International Neuropsychological Society: JINS*. 16:106.
35. Passarotti AM, Sweeney JA, Pavuluri MN (2010): Neural correlates of response inhibition in pediatric bipolar disorder and attention deficit hyperactivity disorder. *Psychiatry Research: Neuroimaging*. 181:36-43.
36. Poissant H, Rapin L, Chenail S, Mendrek A (2016): Forethought in youth with attention deficit/hyperactivity disorder: An FMRI study of sex-specific differences. *Psychiatry journal*. 2016.
37. Posner J, Nagel BJ, Maia TV, Mechling A, Oh M, Wang Z, et al. (2011): Abnormal amygdalar activation and connectivity in adolescents with attention-deficit/hyperactivity disorder. *Journal of the American Academy of Child & Adolescent Psychiatry*. 50:828-837. e823.
38. Posner J, Maia TV, Fair D, Peterson BS, Sonuga-Barke EJ, Nagel BJ (2011): The attenuation of dysfunctional emotional processing with stimulant medication: an fMRI study of adolescents with ADHD. *Psychiatry Research: Neuroimaging*. 193:151-160.
39. Rubia K, Smith AB, Brammer MJ, Toone B, Taylor E (2005): Abnormal brain activation during inhibition and error detection in medication-naive adolescents with ADHD. *American Journal of Psychiatry*. 162:1067-1075.
40. Rubia K, Halari R, Smith AB, Mohammed M, Scott S, Giampietro V, et al. (2008): Dissociated functional brain abnormalities of inhibition in boys with pure conduct disorder and in boys with pure attention deficit hyperactivity disorder. *American Journal of Psychiatry*. 165:889-897.
41. Rubia K, Smith AB, Halari R, Matsukura F, Mohammad M, Taylor E, et al. (2009): Disorder-specific dissociation of orbitofrontal dysfunction in boys with pure conduct disorder during reward and ventrolateral prefrontal dysfunction in boys with pure ADHD during sustained attention. *American Journal of Psychiatry*. 166:83-94.
42. Rubia K, Halari R, Smith AB, Mohammad M, Scott S, Brammer MJ (2009): Shared and disorder-specific prefrontal abnormalities in boys with pure attention-deficit/hyperactivity

disorder compared to boys with pure CD during interference inhibition and attention allocation. *Journal of Child Psychology and Psychiatry*. 50:669-678.
43. Rubia K, Halari R, Christakou A, Taylor E (2009): Impulsiveness as a timing disturbance: neurocognitive abnormalities in attention-deficit hyperactivity disorder during temporal processes and normalization with methylphenidate. *Philosophical Transactions of the Royal Society B: Biological Sciences*. 364:1919-1931.
44. Rubia K, Halari R, Cubillo A, Mohammad AM, Scott S, Brammer M (2010): Disorder-specific inferior prefrontal hypofunction in boys with pure attention-deficit/hyperactivity disorder compared to boys with pure conduct disorder during cognitive flexibility. *Human Brain Mapping*. 31:1823-1833.
45. Rubia K, Cubillo A, Smith AB, Woolley J, Heyman I, Brammer MJ (2010): Disorder-specific dysfunction in right inferior prefrontal cortex during two inhibition tasks in boys with attention-deficit hyperactivity disorder compared to boys with obsessive–compulsive disorder. *Human brain mapping*. 31:287-299.
46. Rubia K, Cubillo A, Woolley J, Brammer MJ, Smith A (2011): Disorder-specific dysfunctions in patients with attention-deficit/hyperactivity disorder compared to patients with obsessive-compulsive disorder during interference inhibition and attention allocation. *Human brain mapping*. 32:601-611.
47. Schwarz NF, Krafft CE, Chi L, Weinberger AL, Schaeffer DJ, Pierce JE, et al. (2015): Antisaccade-related brain activation in children with attention-deficit/hyperactivity disorder–A pilot study. *Psychiatry Research: Neuroimaging*. 234:272-279.
48. Sheridan MA, Hinshaw S, D'ESPOSITO M (2007): Efficiency of the prefrontal cortex during working memory in attention-deficit/hyperactivity disorder. *Journal of the American Academy of Child & Adolescent Psychiatry*. 46:1357-1366.
49. Silk TJ, Vance A, Rinehart N, Bradshaw JL, Cunnington R (2008): Dysfunction in the fronto-parietal network in attention deficit hyperactivity disorder (ADHD): an fMRI study. *Brain Imaging and Behavior*. 2:123-131.
50. Siniatchkin M, Glatthaar N, von Müller GG, Prehn-Kristensen A, Wolff S, Knöchel S, et al. (2012): Behavioural treatment increases activity in the cognitive neuronal networks in children with attention deficit/hyperactivity disorder. *Brain topography*. 25:332-344.
51. Smith AB, Taylor E, Brammer M, Halari R, Rubia K (2008): Reduced activation in right lateral prefrontal cortex and anterior cingulate gyrus in medication-naïve adolescents with attention deficit hyperactivity disorder during time discrimination. *Journal of Child Psychology and Psychiatry*. 49:977-985.
52. Spinelli S, Joel S, Nelson TE, Vasa RA, Pekar JJ, Mostofsky SH (2011): Different neural patterns are associated with trials preceding inhibitory errors in children with and without attention-deficit/hyperactivity disorder. *Journal of the American Academy of Child & Adolescent Psychiatry*. 50:705-715. e703.
53. Spinelli S, Vasa RA, Joel S, Nelson TE, Pekar JJ, Mostofsky SH (2011): Variability in post-error behavioral adjustment is associated with functional abnormalities in the temporal cortex in children with ADHD. *Journal of Child Psychology and Psychiatry*. 52:808-816.
54. Stevens MC, Pearlson GD, Calhoun VD, Bessette KL (2018): Functional neuroimaging evidence for distinct neurobiological pathways in attention-deficit/hyperactivity disorder. *Biological Psychiatry: Cognitive Neuroscience and Neuroimaging*. 3:675-685.
55. Tamm L, Menon V, Ringel J, Reiss AL (2004): Event-related FMRI evidence of frontotemporal involvement in aberrant response inhibition and task switching in attention-

deficit/hyperactivity disorder. *Journal of the American Academy of Child & Adolescent Psychiatry*. 43:1430-1440.
56. Tamm L, Menon V, Reiss AL (2006): Parietal attentional system aberrations during target detection in adolescents with attention deficit hyperactivity disorder: event-related fMRI evidence. *American Journal of Psychiatry*. 163:1033-1043.
57. Tamm L, Juranek J (2012): Fluid reasoning deficits in children with ADHD: Evidence from fMRI. *Brain research*. 1465:48-56.
58. Tegelbeckers J, Bunzeck N, Duzel E, Bonath B, Flechtner HH, Krauel K (2015): Altered salience processing in attention deficit hyperactivity disorder. *Human brain mapping*. 36:2049-2060.
59. Vance A, Silk T, Casey M, Rinehart NJ, Bradshaw JL, Bellgrove MA, et al. (2007): Right parietal dysfunction in children with attention deficit hyperactivity disorder, combined type: a functional MRI study. *Molecular psychiatry*. 12:826-832.
60. van Ewijk H, Weeda WD, Heslenfeld DJ, Luman M, Hartman CA, Hoekstra PJ, et al. (2015): Neural correlates of visuospatial working memory in attention-deficit/hyperactivity disorder and healthy controls. *Psychiatry Research: Neuroimaging*. 233:233-242.
61. van Rooij D, Hoekstra PJ, Mennes M, von Rhein D, Thissen AJ, Heslenfeld D, et al. (2015): Distinguishing adolescents with ADHD from their unaffected siblings and healthy comparison subjects by neural activation patterns during response inhibition. *American Journal of Psychiatry*. 172:674-683.
62. Vetter NC, Buse J, Backhausen LL, Rubia K, Smolka MN, Roessner V (2018): Anterior insula hyperactivation in ADHD when faced with distracting negative stimuli. *Human brain mapping*. 39:2972-2986.
63. Vloet TD, Gilsbach S, Neufang S, Fink GR, Herpertz-Dahlmann B, Konrad K (2010): Neural mechanisms of interference control and time discrimination in attention-deficit/hyperactivity disorder. *Journal of the American Academy of Child & Adolescent Psychiatry*. 49:356-367.
64. von Rhein D, Cools R, Zwiers MP, van der Schaaf M, Franke B, Luman M, et al. (2015): Increased neural responses to reward in adolescents and young adults with attention-deficit/hyperactivity disorder and their unaffected siblings. *Journal of the American Academy of Child & Adolescent Psychiatry*. 54:394-402.
65. Wang S, Yang Y, Xing W, Chen J, Liu C, Luo X (2013): Altered neural circuits related to sustained attention and executive control in children with ADHD: an event-related fMRI study. *Clinical Neurophysiology*. 124:2181-2190.
66. Hegarty CE, Gupta MW, Miller E, Terashima K, Loo S, McCracken J, et al. (2019): Symptom severity in youths with attention deficit hyperactivity disorder associated with normalizing effects of treatment on fMRI response during a stop signal task. *bioRxiv*.599803.
67. Finger EC, Marsh AA, Mitchell DG, Reid ME, Sims C, Budhani S, et al. (2008): Abnormal ventromedial prefrontal cortex function in children with psychopathic traits during reversal learning. *Archives of general psychiatry*. 65:586-594.
68. Gatzke-Kopp LM, Beauchaine TP, Shannon KE, Chipman J, Fleming AP, Crowell SE, et al. (2009): Neurological correlates of reward responding in adolescents with and without externalizing behavior disorders. *Journal of abnormal psychology*. 118:203.
69. Bubenzer-Busch S, Herpertz-Dahlmann B, Kuzmanovic B, Gaber T, Helmbold K, Ullisch M, et al. (2016): Neural correlates of reactive aggression in children with attention-

deficit/hyperactivity disorder and comorbid disruptive behaviour disorders. *Acta Psychiatrica Scandinavica*. 133:310-323.
70. Banich MT, Crowley TJ, Thompson LL, Jacobson BL, Liu X, Raymond KM, et al. (2007): Brain activation during the Stroop task in adolescents with severe substance and conduct problems: A pilot study. *Drug and Alcohol Dependence*. 90:175-182.
71. Bjork JM, Chen G, Smith AR, Hommer DW (2010): Incentive-elicited mesolimbic activation and externalizing symptomatology in adolescents. *Journal of Child Psychology and Psychiatry*. 51:827-837.
72. Crowley TJ, Dalwani MS, Mikulich-Gilbertson SK, Du YP, Lejuez CW, Raymond KM, et al. (2010): Risky decisions and their consequences: neural processing by boys with antisocial substance disorder. *PloS one*. 5:e12835.
73. Dong D, Ming Q, Wang X, Yu W, Jiang Y, Wu Q, et al. (2017): Temporoparietal junction hypoactivity during pain-related empathy processing in adolescents with conduct disorder. *Frontiers in psychology*. 7:2085.
74. Fairchild G, Baker E, Eaton S (2018): Hypothalamic-pituitary-adrenal axis function in children and adults with severe antisocial behavior and the impact of early adversity. *Current psychiatry reports*. 20:84.
75. Fehlbaum LV, Raschle NM, Menks WM, Prätzlich M, Flemming E, Wyss L, et al. (2018): Altered neuronal responses during an affective Stroop task in adolescents with conduct disorder. *Frontiers in psychology*. 9:1961.
76. Finger EC, Marsh AA, Blair KS, Reid ME, Sims C, Ng P, et al. (2011): Disrupted reinforcement signaling in the orbitofrontal cortex and caudate in youths with conduct disorder or oppositional defiant disorder and a high level of psychopathic traits. *American Journal of Psychiatry*. 168:152-162.
77. Herpertz SC, Huebner T, Marx I, Vloet TD, Fink GR, Stoecker T, et al. (2008): Emotional processing in male adolescents with childhood-onset conduct disorder. *Journal of Child Psychology and Psychiatry*. 49:781-791.
78. Hwang S, Nolan ZT, White SF, Williams WC, Sinclair S, Blair R (2016): Dual neurocircuitry dysfunctions in disruptive behavior disorders: emotional responding and response inhibition. *Psychological medicine*. 46:1485-1496.
79. Klapwijk ET, Aghajani M, Colins OF, Marijnissen GM, Popma A, van Lang ND, et al. (2016): Different brain responses during empathy in autism spectrum disorders versus conduct disorder and callous-unemotional traits. *Journal of Child Psychology and Psychiatry*. 57:737-747.
80. Klapwijk ET, Lelieveld G-J, Aghajani M, Boon AE, van der Wee NJ, Popma A, et al. (2016): Fairness decisions in response to emotions: a functional MRI study among criminal justice-involved boys with conduct disorder. *Social cognitive and affective neuroscience*. 11:674-682.
81. Marsh AA, Finger EC, Fowler KA, Jurkowitz IT, Schechter JC, Henry HY, et al. (2011): Reduced amygdala–orbitofrontal connectivity during moral judgments in youths with disruptive behavior disorders and psychopathic traits. *Psychiatry Research: Neuroimaging*. 194:279-286.
82. Marsh AA, Finger EC, Fowler KA, Adalio CJ, Jurkowitz IT, Schechter JC, et al. (2013): Empathic responsiveness in amygdala and anterior cingulate cortex in youths with psychopathic traits. *Journal of child psychology and psychiatry*. 54:900-910.
83. Passamonti L, Fairchild G, Goodyer IM, Hurford G, Hagan CC, Rowe JB, et al. (2010): Neural abnormalities in early-onset and adolescence-onset conduct disorder. *Archives of general psychiatry*. 67:729-738.

84. Schwenck C, Ciaramidaro A, Selivanova M, Tournay J, Freitag CM, Siniatchkin M (2017): Neural correlates of affective empathy and reinforcement learning in boys with conduct problems: fMRI evidence from a gambling task. *Behavioural brain research*. 320:75-84.
85. Thornton LC, Penner EA, Nolan ZT, Adalio CJ, Sinclair S, Meffert H, et al. (2017): The processing of animacy information is disrupted as a function of callous-unemotional traits in youth with disruptive behavior disorders. *NeuroImage: Clinical*. 16:498-506.
86. White SF, Williams WC, Brislin SJ, Sinclair S, Blair KS, Fowler KA, et al. (2012): Reduced activity within the dorsal endogenous orienting of attention network to fearful expressions in youth with disruptive behavior disorders and psychopathic traits. *Development and psychopathology*. 24:1105-1116.
87. White SF, Marsh AA, Fowler KA, Schechter JC, Adalio C, Pope K, et al. (2012): Reduced amygdala response in youths with disruptive behavior disorders and psychopathic traits: decreased emotional response versus increased top-down attention to nonemotional features. *American Journal of Psychiatry*. 169:750-758.
88. White SF, Pope K, Sinclair S, Fowler KA, Brislin SJ, Williams WC, et al. (2013): Disrupted expected value and prediction error signaling in youths with disruptive behavior disorders during a passive avoidance task. *American Journal of Psychiatry*. 170:315-323.
89. White SF, Fowler KA, Sinclair S, Schechter JC, Majestic CM, Pine DS, et al. (2014): Disrupted expected value signaling in youth with disruptive behavior disorders to environmental reinforcers. *Journal of the American Academy of Child & Adolescent Psychiatry*. 53:579-588. e579.
90. Zhang J, Li B, Gao J, Shi H, Wang X, Jiang Y, et al. (2015): Impaired frontal-basal ganglia connectivity in male adolescents with conduct disorder. *PloS one*. 10:e0145011.
91. Zhu Y, Ying K, Wang J, Su L, Chen J, Lin F, et al. (2014): Differences in functional activity between boys with pure oppositional defiant disorder and controls during a response inhibition task: a preliminary study. *Brain imaging and behavior*. 8:588-597.
92. Raschle NM, Fehlbaum LV, Menks WM, Martinelli A, Prätzlich M, Bernhard A, et al. (2019): Atypical dorsolateral prefrontal activity in female adolescents with conduct disorder during effortful emotion regulation. *Biological Psychiatry: Cognitive Neuroscience and Neuroimaging*. 4:984-994.
93. Yang P, Wu M-T, Hsu C-C, Ker J-H (2004): Evidence of early neurobiological alternations in adolescents with posttraumatic stress disorder: a functional MRI study. *Neuroscience letters*. 370:13-18.
94. Carrion VG, Garrett A, Menon V, Weems CF, Reiss AL (2008): Posttraumatic stress symptoms and brain function during a response-inhibition task: an fMRI study in youth. *Depression and anxiety*. 25:514-526.
95. Keding TJ, Herringa RJ (2016): Paradoxical prefrontal–amygdala recruitment to angry and happy expressions in pediatric posttraumatic stress disorder. *Neuropsychopharmacology*. 41:2903-2912.
96. Hart H, Lim L, Mehta M, Simmons A, Mirza K, Rubia K (2018): Altered fear processing in adolescents with a history of severe childhood maltreatment: an fMRI study. *Psychological medicine*. 48:1092-1101.
97. Carlisi CO, Hilbert K, Guyer AE, Ernst M (2017): Sleep-amount differentially affects fear-processing neural circuitry in pediatric anxiety: a preliminary fMRI investigation. *Cognitive, Affective, & Behavioral Neuroscience*. 17:1098-1113.

98. Gold AL, Abend R, Britton JC, Behrens B, Farber M, Ronkin E, et al. (2020): Age differences in the neural correlates of anxiety disorders: An fMRI study of response to learned threat. *American Journal of Psychiatry*. 177:454-463.
99. Thomas KM, Drevets WC, Dahl RE, Ryan ND, Birmaher B, Eccard CH, et al. (2001): Amygdala response to fearful faces in anxious and depressed children. *Archives of general psychiatry*. 58:1057-1063.
100. Monk CS, Nelson EE, McClure EB, Mogg K, Bradley BP, Leibenluft E, et al. (2006): Ventrolateral prefrontal cortex activation and attentional bias in response to angry faces in adolescents with generalized anxiety disorder. *American Journal of Psychiatry*. 163:1091-1097.
101. Monk CS, Telzer EH, Mogg K, Bradley BP, Mai X, Louro HM, et al. (2008): Amygdala and ventrolateral prefrontal cortex activation to masked angry faces in children and adolescents with generalized anxiety disorder. *Archives of general psychiatry*. 65:568-576.
102. Strawn JR, Bitter SM, Weber WA, Chu WJ, Whitsel RM, Adler C, et al. (2012): Neurocircuitry of generalized anxiety disorder in adolescents: a pilot functional neuroimaging and functional connectivity study. *Depression and Anxiety*. 29:939-947.
103. Yin D, Liu W, Zeljic K, Lv Q, Wang Z, You M, et al. (2017): Failure in cognitive suppression of negative affect in adolescents with generalized anxiety disorder. *Scientific reports*. 7:1-11.
104. Burkhouse KL, Kujawa A, Hosseini B, Klumpp H, Fitzgerald KD, Langenecker SA, et al. (2018): Anterior cingulate activation to implicit threat before and after treatment for pediatric anxiety disorders. *Progress in Neuro-Psychopharmacology and Biological Psychiatry*. 84:250-256.
105. Blair KS, Geraci M, Korelitz K, Otero M, Towbin K, Ernst M, et al. (2011): The pathology of social phobia is independent of developmental changes in face processing. *American Journal of Psychiatry*. 168:1202-1209.
106. Chantiluke K, Halari R, Simic M, Pariante CM, Papadopoulos A, Giampietro V, et al. (2012): Fronto-striato-cerebellar dysregulation in adolescents with depression during motivated attention. *Biological psychiatry*. 71:59-67.
107. Colich NL, Foland-Ross LC, Eggleston C, Singh MK, Gotlib IH (2016): Neural aspects of inhibition following emotional primes in depressed adolescents. *Journal of Clinical Child & Adolescent Psychology*. 45:21-30.
108. Davey CG, Allen NB, Harrison BJ, Yücel M (2011): Increased amygdala response to positive social feedback in young people with major depressive disorder. *Biological psychiatry*. 69:734-741.
109. Diler RS, de Almeida JRC, Ladouceur C, Birmaher B, Axelson D, Phillips M (2013): Neural activity to intense positive versus negative stimuli can help differentiate bipolar disorder from unipolar major depressive disorder in depressed adolescents: a pilot fMRI study. *Psychiatry Research: Neuroimaging*. 214:277-284.
110. Diler RS, Pan LA, Segreti A, Ladouceur CD, Forbes E, Cela SR, et al. (2014): Differential anterior cingulate activity during response inhibition in depressed adolescents with bipolar and unipolar major depressive disorder. *Journal of the Canadian Academy of Child and Adolescent Psychiatry*. 23:10.
111. Gaffrey MS, Barch DM, Singer J, Shenoy R, Luby JL (2013): Disrupted amygdala reactivity in depressed 4-to 6-year-old children. *Journal of the American Academy of Child & Adolescent Psychiatry*. 52:737-746.

112. Halari R, Simic M, Pariante CM, Papadopoulos A, Cleare A, Brammer M, et al. (2009): Reduced activation in lateral prefrontal cortex and anterior cingulate during attention and cognitive control functions in medication-naïve adolescents with depression compared to controls. *Journal of Child Psychology and Psychiatry*. 50:307-316.
113. Hall LM, Klimes-Dougan B, Hunt RH, Thomas KM, Houri A, Noack E, et al. (2014): An fMRI study of emotional face processing in adolescent major depression. *Journal of affective disorders*. 168:44-50.
114. Roberson-Nay R, McClure EB, Monk CS, Nelson EE, Guyer AE, Fromm SJ, et al. (2006): Increased amygdala activity during successful memory encoding in adolescent major depressive disorder: an fMRI study. *Biological psychiatry*. 60:966-973.
115. Sharp C, Kim S, Herman L, Pane H, Reuter T, Strathearn L (2014): Major depression in mothers predicts reduced ventral striatum activation in adolescent female offspring with and without depression. *Journal of abnormal psychology*. 123:298.
116. Tao R, Calley CS, Hart J, Mayes TL, Nakonezny PA, Lu H, et al. (2012): Brain activity in adolescent major depressive disorder before and after fluoxetine treatment. *American Journal of Psychiatry*. 169:381-388.
117. Yang TT, Simmons AN, Matthews SC, Tapert SF, Frank GK, Bischoff-Grethe A, et al. (2009): Depressed adolescents demonstrate greater subgenual anterior cingulate activity. *Neuroreport*. 20:440.
118. Yang TT, Simmons AN, Matthews SC, Tapert SF, Frank GK, Max JE, et al. (2010): Adolescents with major depression demonstrate increased amygdala activation. *Journal of the American Academy of Child & Adolescent Psychiatry*. 49:42-51.
119. Pan LA, Batezati-Alves SC, Almeida JR, Segreti A, Akkal D, Hassel S, et al. (2011): Dissociable patterns of neural activity during response inhibition in depressed adolescents with and without suicidal behavior. *Journal of the American Academy of Child & Adolescent Psychiatry*. 50:602-611. e603.
120. Pan L, Segreti A, Almeida J, Jollant F, Lawrence N, Brent D, et al. (2013): Preserved hippocampal function during learning in the context of risk in adolescent suicide attempt. *Psychiatry Research: Neuroimaging*. 211:112-118.
121. Groschwitz RC, Plener PL, Groen G, Bonenberger M, Abler B (2016): Differential neural processing of social exclusion in adolescents with non-suicidal self-injury: An fMRI study. *Psychiatry Research: Neuroimaging*. 255:43-49.
122. Suzuki H, Luby JL, Botteron KN, Dietrich R, McAvoy MP, Barch DM (2014): Early life stress and trauma and enhanced limbic activation to emotionally valenced faces in depressed and healthy children. *Journal of the American Academy of Child & Adolescent Psychiatry*. 53:800-813. e810.
123. De Bellis MD, Hooper SR (2012): Neural substrates for processing task-irrelevant emotional distracters in maltreated adolescents with depressive disorders: A pilot study. *Journal of traumatic stress*. 25:198-202.
